# Proinflammatory macrophages transporting gut-derived bacterial DNA drive autoimmune arthritis in spondyloarthropathy

**DOI:** 10.1172/jci.insight.188028

**Published:** 2025-07-31

**Authors:** Benjamin Cai, Rabina Giri, Amy J. Cameron, M. Arifur Rahman, Annabelle Small, Christopher Altmann, Yenkai Lim, Linda M. Rehaume, Mark Morrison, Mihir D. Wechalekar, Jakob Begun, Anne-Sophie Bergot, Ranjeny Thomas

**Affiliations:** 1 Frazer Institute, Faculty of Health, Medicine and Behavioural Sciences, and; 2 Mater Research Institute, The University of Queensland, Brisbane, Australia.; 3 College of Medicine & Public Health, Flinders University and Flinders Medical Centre, Bedford Park, South Australia, Australia.

**Keywords:** Autoimmunity, Inflammation, Arthritis, Autoimmune diseases

## Abstract

Spondyloarthritis (SpA) is an inflammatory arthritis of the spine and joints associated with intestinal inflammation, in which it is hypothesized that innate immune exposure to enteroinvasive species is followed by self-/bacterial peptide presentation. However, the mechanisms underlying loss of tolerance to gut bacteria in genetically at-risk individuals are unclear. Curdlan-treated (β-1,3-glucan, dectin-1 ligand–treated) ZAP-70^W163C^ (SKG) mice develop autoimmune arthritis and ileitis associated with Gram-negative fecal dysbiosis. Using gnotobiotic mice, we show that curdlan-treated SKG mice monoassociated with *Parabacteroides goldsteinii* or *Lactobacillus murinus* developed ileitis, arthritis, and enthesitis, while BALB/c mice were tolerant. Gnotobiotic SKG ileum upregulated *Il23a* and ER stress genes and lost goblet cells. Whereas bacterial DNA colocalized with neutrophils and inflammatory macrophages in SKG lamina propria, periarticular bone marrow, entheses, and spleen, in BALB/c mice, bacterial DNA colocalized with resident macrophages in lamina propria and spleen. Human psoriatic-arthritis synovial tissue also contained cell-associated perivascular bacterial DNA. Curdlan-treated SKG spleen/bone marrow macrophages transferred severe arthritis and expanded Th17 cells in naive SKG recipients, while BALB/c or germ-free SKG macrophages transferred mild arthritis and regulated Th17 cells. Thus, bacterial DNA and myeloid cells in the gut and their subsequent traffic regulate or enforce T cell pathogenicity in SpA.

## Abstract 2

Patients with spondyloarthritis have intestinal inflammation. In genetically-predisposed mice, inflammatory macrophages traffic bacterial DNA to expand autoreactive Th17, disrupting regulation by commensal DNA-associated resident macrophages

## Graphical abstract

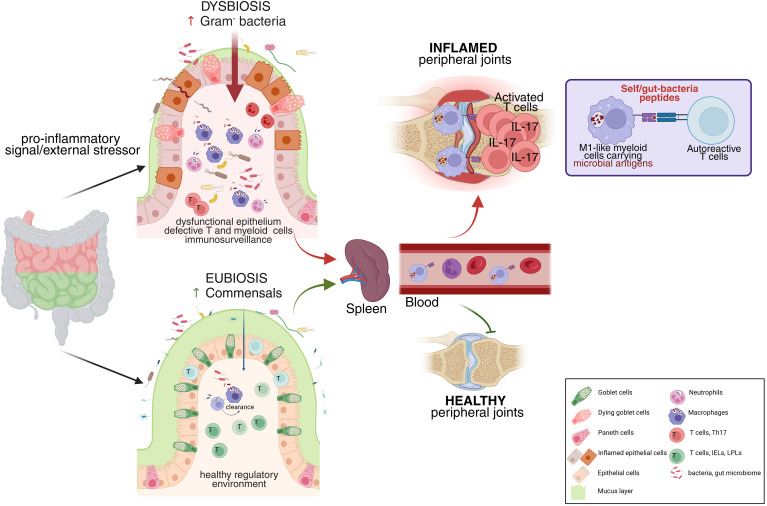

## Introduction

Spondyloarthropathies (SpA) are chronic rheumatic inflammatory diseases, including *HLA-B27*–associated ankylosing spondylitis (AS, axial SpA), psoriatic arthritis (PsA), and reactive arthritis (ReA), that affect 1%–2% of the population. Up to 70% of patients have evidence of extra-articular gut inflammation, and genome-wide association studies have identified high proportions of shared associated genetic loci with inflammatory bowel disease (IBD) (1, 2), highlighting the relevance of gut inflammation to disease pathogenesis (3).

Gut microbial dysbiosis in SpA rodent models, such as ZAP70^W163C^ mutant (SKG) mice on a BALB/c background, and patients (4) is often associated with disease activity (5, 6). SKG mice have reduced T cell receptor (TCR) signal strength and thymic selection (7, 8), functionally impaired Foxp3^+^ Tregs, Th17-skewed autoreactive CD4^+^ T cells, mild systemic CD4 and CD8 T lymphopenia (9) and depleted ileal cytotoxic T cells, reflecting T cell immunodeficiency for bacterial control and dysfunctional inflammatory regulation. These features are also observed in human AS (10, 11). Similar to patients with SpA (12), naive SKG mice have intestinal Gram-negative dysbiosis, associated with increased *Prevotellaceae*, *Bacteroidaceae*, and *Porphyromonadaceae* (essentially as *Parabacteroides spp.*), with constitutive ileal expression of *Il23a* and endoplasmic reticulum (ER) stress, characteristics that are reversible with anti–IL-23p19 treatment and expansion of homeostasis-inducing microbiota, including *Lactobacillaceae* (13–15). After systemic administration of microbial β-1,3-glucan (curdlan), SKG mice raised in specific pathogen–free (SPF) but not germ-free (GF) conditions develop IL-23/IL-22/IL-17 axis-dependent arthritis of spine and joints, psoriasiform skin inflammation and ileal goblet cell loss, gut barrier disruption, and ileitis (9, 13–15). However, the interplay between pathobiont and homeostatic species that leads to IL-23–dependent dysbiosis in SKG but not BALB/c hosts is not well understood.

Although expanded *Parabacteroides spp*. correlated with disease severity in SKG mice (14), *Parabacteroides distasonis* and microbial-derived bile acids were protective in collagen-induced arthritis and autoimmune-prone TNF transgenic mice (16). In immunocompetent models, *Parabacteroides goldsteinii* (17–20) and *Lactobacillus spp.* (21–24) are often associated with antiinflammatory properties and disease mitigation, which would define them as potential probiotic candidates (17). Thus, it is unclear why these homeostatic-inducing bacteria fail to protect SKG mice from ileitis and arthritis.

To elucidate this, we colonized GF SKG or BALB/c mice with single gut bacterial species or the altered Schaedler flora (ASF) miniconsortium (15) to assess their contribution to the pathogenesis of SpA. We show that after curdlan, arthritis, enthesitis, and ileitis severity in SKG hosts depend on the colonizing bacteria, IL-23, and the phenotype and function of the bacterial DNA-associated myeloid antigen-presenting cells stimulating autoreactive T cells. Furthermore, hematogenous spread of nonviable bacterial DNA carried by neutrophils and proinflammatory macrophages from the gut to spleen, bone marrow, and entheses promotes CD4^+^ Th17 expansion with reduced regulation, joint T cell infiltration, and severe arthritis.

## Results

### Gut bacteria are necessary for IL-23–dependent curdlan-induced ileitis and arthritis, but not enthesitis, in monoassociated SKG mice.

**Figure 1:**
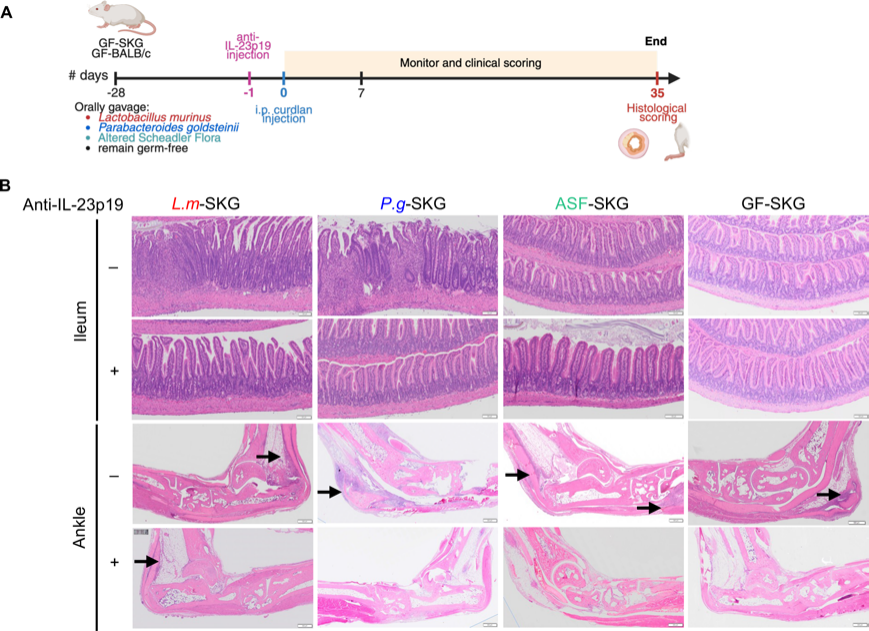
Monoassociations with *P*. *goldsteinii* and *L*. *murinus* are sufficient for curdlan-induced IL-23–dependent ileitis and arthritis. GF-SKG mice were monocolonized with *L*. *murinus*, *P*. *goldsteinii* or ASF 4 weeks prior to curdlan i.p. at day 0, with *n* = 4–9 across 3 independent experiments. Some mice were treated with anti–IL-23p19 (60 μg, i.p.) at day –1. Mice were monitored for 5 weeks. (**A**) Experiment design. (**B**) Representative H&E staining of ileum and rear ankle. Scale bars: 100 μm (top) and 500 μm (bottom). Arrows point to areas of immune infiltration. Magnified images for the rear ankle are shown in Supplemental Figure 1.

**Figure 2:**
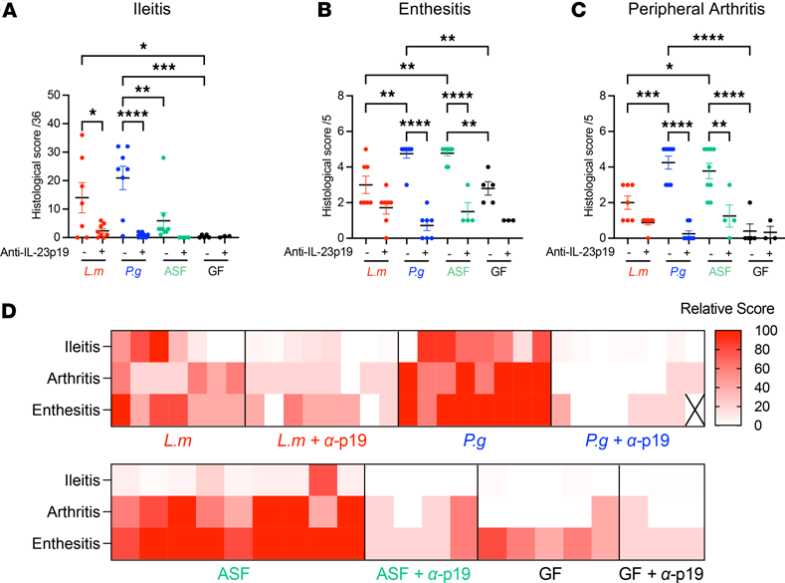
Monoassociations with *P*. *goldsteinii* and *L*. *murinus* are sufficient for curdlan-induced IL-23–dependent ileitis and arthritis. GF-SKG mice were monocolonized with *L*. *murinus*, *P*. *goldsteinii*, or ASF 4 weeks prior to curdlan i.p. at day 0, with *n* = 4–9 across 3 independent experiments. Some mice were treated with anti–IL-23p19 (60 μg, i.p.) at day –1. Mice were monitored for 5 weeks. (**A**–**C**) Histological sections were scored blindly and cumulative score at 5 weeks after curdlan of ileitis (**A**), enthesitis (**B**), and peripheral arthritis (**C**). (**D**) Heatmap of combined ileitis, enthesitis, and peripheral arthritis histological scores per mice per group. Data show mean ± SEM, with each data point representing an individual mouse. Two-way ANOVA (**C**–**E**) with *P < 0.5, **P < 0.01, ***P < 0.001, ****P < 0.0001.

Relative to BALB/c mice, SKG mice have a dysbiotic gut microbiota with increased Gram-negative bacteria that reverses with anti–IL-23p19 treatment (14). Within 1 week of systemic curdlan administration, goblet cells are depleted in the ileum of SKG mice (14), followed by the development of ileitis and spondyloarthritis (9). To directly demonstrate pathogenicity of a single species and its mechanism, GF-SKG or GF-BALB/c mice were orally gavaged with *P*. *goldsteinii* (*P.g.*), *L*. *murinus* (*L.m.*), the ASF miniconsortium (*P.g*., *L.m*., *Clostridium sp.*, and *M*. *schaedleri*) (15) or no bacteria, followed by i.p. curdlan 4 weeks later. Some groups were administered anti–IL-23p19 one day prior to curdlan (Figure 1A). Tissues were scored histologically 5 weeks later (Figure 1B and Supplemental Figure 1; supplemental material available online with this article; https://doi.org/10.1172/jci.insight.188028DS1). Gnotobiotic BALB/c mice were resistant to curdlan-induced disease (Supplemental Figure 2). *P.g*. or *L.m*. monoassociation and curdlan induced ileitis (Figure 1B and Figure 2A). *P.g*. monoassociation induced enthesitis (Figure 1Β and Figure 2B) and peripheral arthritis (Figure 1B and Figure 2C) that were significantly more severe than in curdlan-treated GF-SKG mice. All 3 pathologies were IL-23 dependent. After *L.m*. monoassociation, peripheral arthritis and enthesitis were significantly milder than in *P.g*. monoassociated mice (Figure 2, A–D).

Although GF-SKG mice were resistant to ileitis and peripheral arthritis (Figure 1B and Figure 2, A and C), they developed Achilles enthesitis after curdlan (Figure 1B and Figure 2B), consistent with previously reported mild enthesitis involving spinal ligaments in curdlan-treated GF SKG mice (15). Enthesitis in GF-SKG mice was significantly less severe than in *P.g*. monoassociated SKG mice, highlighting the aggravating role of *P.g*. ASF-colonized SKG mice developed IL-23p19-dependent arthritis and enthesitis of similar relative intensity to *P.g*.-SKG mice but were protected from ileitis (Figure 1B and Figure 2, A–D).

Thus, after curdlan, mice colonized with *P.g*. alone or *P.g*. within the ASF miniconsortium developed severe ileitis, paw inflammation characterized by IL-23–dependent enthesitis and peripheral arthritis. Mice colonized with *L.m*. developed ileitis, mild arthritis and mild enthesitis, and GF mice developed mild enthesitis (relative severities shown in Figure 2D). These data indicate that gut bacteria were necessary for the development of curdlan-induced peripheral arthritis and ileitis in SKG but not BALB/c mice. Therefore, both *P.g*. and *L.m*. acted as pathobionts in SKG mice, but the effects of *P.g*. monoassociation were more severe and more consistently IL-23p19 dependent than those of *L*.*m*. monoassociation.

To explore the relative abundance of each of the 4 bacteria in the ASF bacterial consortium in response to curdlan, we colonized GF mice with ASF and treated with either saline or curdlan, with or without anti–IL-23p19 or isotype control mAb. We then quantified the DNA copies of each species by quantitative PCR (qPCR) in fecal pellets at days 5, 7, 14, 35, and 56 after curdlan relative to the precurdlan level, as an indication of the abundance of each species over time. While *P.g.*, *M*. *Schaedleri*, and *Clostridium* *sp*. did not change over time, *L.m*. significantly expanded at day 7 after curdlan in anti–IL-23p19–treated ASF-SKG (Supplemental Figure 3), suggesting that curdlan-induced IL-23 restrains *L.m*. growth.

### Curdlan induced ileal Il23a and ER stress in monoassociated SKG mice.

**Figure 3:**
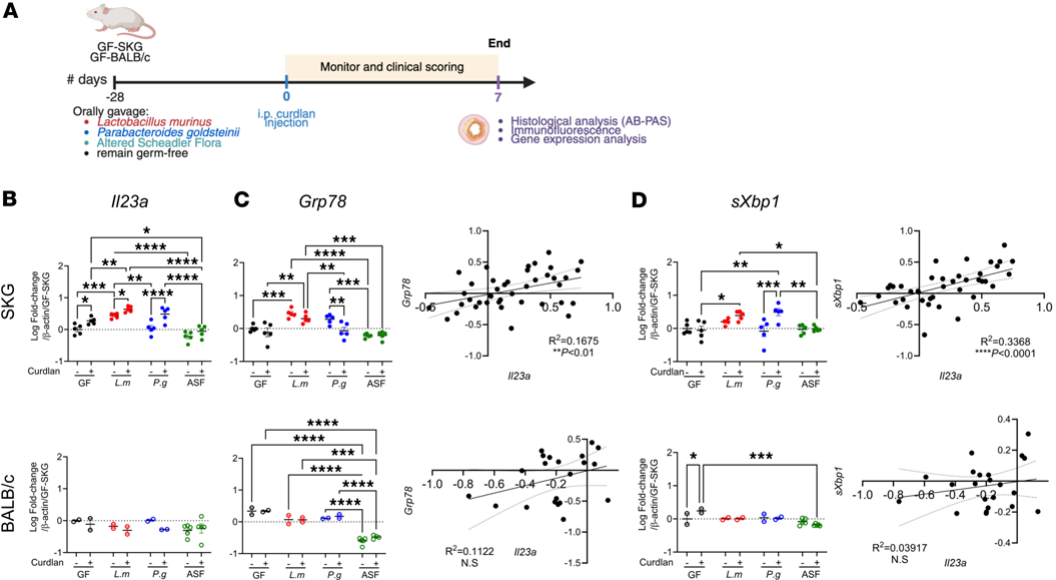
*P*. *goldsteinii* and *L*. *murinus* promote ileal IL-23p19 expression and ER stress after curdlan in gnotobiotic SKG mice. GF-SKG and GF-BALB/c mice were monocolonized with *L.m*., *P.g*., or ASF 4 weeks prior to curdlan i.p. at day 0, with *n* = 4–5 across 2 independent experiments. Mice were sacrificed at day 7, and ileal tissues were assessed for gene expression by qPCR. (**A**) Experiment design for Figures 3–5. (**B**–**D**) Data show the log value of the fold increase against naive GF-SKG. Expression of ER stress markers *Il23a* (**B**), *Grp78* and its correlation to *Il23a* (**C**), and *sXbp1* and its correlation to *Il23a* (**D**). Two-way ANOVA (**B**–**D**) with *P < 0.5, **P < 0.01, ***P < 0.001, ****P < 0.0001.

Since ileitis developed in SKG mice after either *P.g*. or *L.m*. monoassociation but not ASF, we next compared how *P.g*., *L.m*., or the ASF miniconsortium influence ileal inflammation, ER stress, and integrity in SKG and BALB/c mice without or 7 days after curdlan (Figure 3A).

We first compared the expression of *Il23a* and ER stress genes in SKG ileum. In the absence of curdlan and relative to GF ileum, *Il23a* and *Grp78* gene expression significantly increased in mice colonized with *L.m*. but not *P.g*. or ASF (Figure 3, B and C), suggesting a basal ER stress response in the presence of *L.m*. Consistent with the role of IL-23p19 in ileitis (Figure 1B and Figure 2A) and of the relationship of IL-23p19 to ER stress (9, 13–15), curdlan administration increased *Il23a* and *sXbp1* expression in mice colonized with *L.m*. and *P.g*., but not ASF (Figure 3, B and C). In contrast, in ASF-colonized ileum, *Il10* and *Il6* genes were increased (Supplemental Figure 4, A and B), while ER stress–associated *Il23a*, *Grp78*, and s*Xbp1* were not (Figure 3D), supporting a more regulatory environment. In all SKG mice combined, ileal *Il23a* gene expression significantly correlated with *Grp78* and *sXbp1* gene expression (Figure 3, C and D).

In the ileum of *P.g*. or *L.m*. monoassociated BALB/c mice, the expression of *IL23a,* *Grp78*, and *sXbp1* was not different to that of GF mice, and ileum of ASF-BALB/c mice expressed lower levels of *Grp78* than GF or monoassociated mice. In all BALB/c mice combined, ileal *Il23a* gene expression was not correlated with *Grp78* and *sXbp1* gene expression (Figure 3, C and D). GF-SKG had a greater intrinsic proinflammatory potential than GF-BALB/c mice after curdlan, with increased *Il23a*, *Il6*, and *Cxcl2* expression compared with naive GF mice (Figure 3B and Supplemental Figure 4, A–C). These data confirm that the level of *Il23a* is correlated to ER stress in gnotobiotic SKG mice as described in SPF animals (9, 13–15).

### Gut bacteria and curdlan promote loss of ileal integrity in gnotobiotic SKG mice.

**Figure 4:**
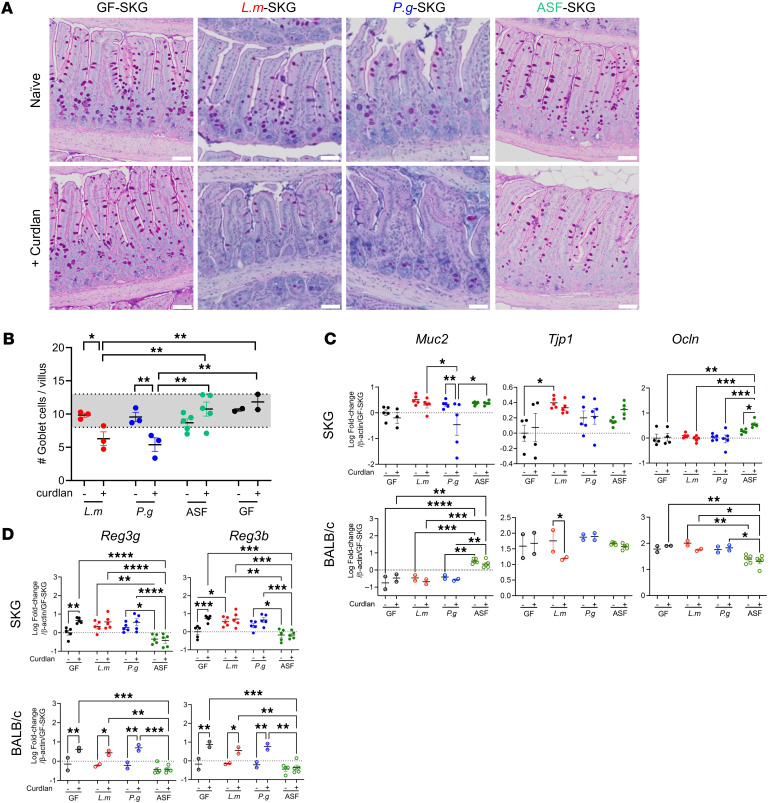
Gut integrity is altered in germ-free and monoassociated SKG mice. GF-SKG and GF-BALB/c mice were orally monocolonized with *L.m*., *P.g*., or ASF or remained GF 4 weeks prior to curdlan i.p. at day 0. Mice were sacrificed at day 7, with *n* = 2–5 across 2 experiments, per experimental design in Figure 3A. (**A**) Representative histology images of ileum stained with PAS/Alcian blue. Scale bar: 50 μm. (**B**) Count of goblet cells per villi. (**C** and **D**) Data show mean ± SEM for the log values of gene expression by qPCR of *Muc2*, *Tjp1*, and *Ocln* (**C**), as well as *Reg3g* and *Reg3b* (**D**), with each data point representing an individual mouse. Two-way ANOVA (**B**–**D**) with *P < 0.5, **P < 0.01, ***P < 0.001, ****P < 0.0001.

Since ileal ER stress in gnotobiotic SKG mice suggests a loss of epithelial permeability and barrier function after curdlan (25), we next compared mucin-producing goblet cells and intercellular tight junction genes, including ZO, claudins, and occludin (26, 27) in the ileum (Figure 3A). To quantify goblet cells, ileal sections were stained with Alcian blue/PAS. Compared with gnotobiotic or GF ileum, goblet cell numbers significantly decreased 7 days after curdlan in SKG mice colonized with *P.g*. or *L.m.* but not ASF (Figure 4, A and B). These data confirm loss of goblet cells, suggesting a loss of gut epithelial integrity, in monoassociated SKG mice, as described in SPF animals (13). SKG ileum colonized with *L.m*. but not *P.g*. expressed higher *Tjp1* (encoding the protein tight junction protein 1 also known as zonula occludens-1 [ZO-1]) than in GF mice (Figure 4C). There were no changes in expression of *Tjp1*, *Ocln* (encoding Occludin), *Cldn* (encoding claudins), or *Reg3g* and *Reg3b* (encoding Paneth cell regenerating islet-derived antimicrobial Reg proteins) in response to curdlan in monoassociated SKG mice (26, 27). However, expression levels of these genes were lower overall in SKG than BALB/c ileum (Figure 4, C and D, and Supplemental Figure 5A). ASF-SKG ileum expressed higher levels of *Ocln* and lower *Reg3g* and *Reg3b* than GF or monoassociated SKG ileum (Figure 4, C and D), consistent with their lack of ileitis and lower ER stress and *Il23a* levels.

**Figure 5:**
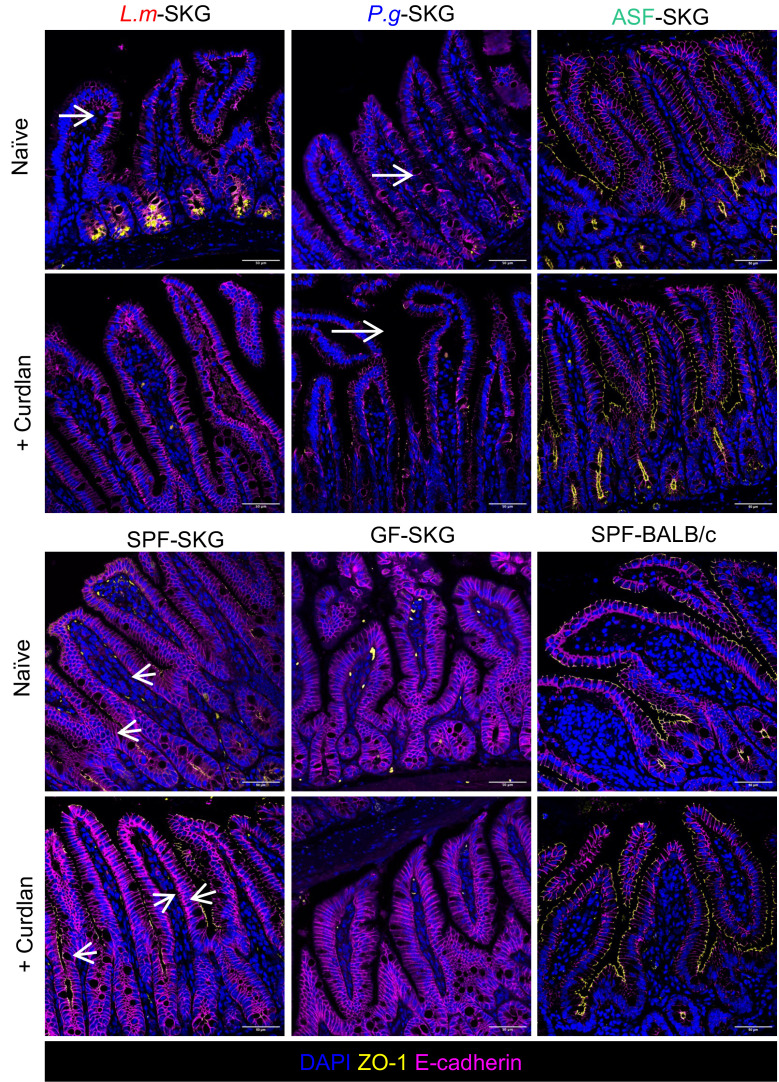
Gut tight junction protein ZO-1 is altered in germ-free and monoassociated SKG mice. GF-SKG and GF-BALB/c mice were orally monocolonized with *L.m*., *P.g*., or ASF or remained GF 4 weeks prior to curdlan i.p. at day 0. SPF-SKG and SPF-BALB/c mice injected with curdlan were used as the controls. Mice were sacrificed at day 7, with *n* = 2–5 across 2 experiments, per experimental design in Figure 3A. Representative histology images of ileal biopsy stained fluorescently with E-cadherin (magenta) and ZO-1 (yellow) obtained by confocal imaging, with *n* = 2–4 across 2 independent staining experiments. Arrows point to ZO-1^+^ staining on the apical surface of the epithelium. Scale bar: 50 µm.

To identify ZO-1^+^ tight junctions among epithelial cells, we stained ileal and control colon tissue sections with ZO-1 and E-cadherin antibodies (Figure 5 and Supplemental Figure 5B). In colon control tissues from naive mice housed in SPF conditions, ZO-1 lined the epithelial cells of BALB/c mice, and there was no loss of integrity with curdlan. In contrast, in naive SPF SKG colon controls, ZO-1 expression was lost after curdlan (Supplemental Figure 5B). ZO-1 was barely expressed in the colon of GF SKG mice (Supplemental Figure 5B) and was only partially restored in *L.m*.-monoassociated naive mice. In the ileum, ZO-1 was sparse in GF, *L.m*.-, and *P.g*.-monoassociated SKG mice, while ZO-1 expression in ASF-colonized SKG mice was similar to that of BALB/c controls, consistent with enhanced tight junction formation with the more diverse miniconsortium (Figure 5).

Since the epithelial contact with *L.m*. but not *P.g*. stimulated *Tjp1* expression in SKG colon in vivo, we cultured the colonic Caco-2 cell line with *L.m*. or *P.g.* to compare their effect on ZO-1 and mucin production. After 4 hours in contact with *L.m*., epithelial cell mucin (stained with WGA-1) and ZO-1 expression increased, whereas *P.g*. did not affect mucin or ZO-1, comparable with the effect of culture medium alone (Supplemental Figure 5C).

Together these data indicate that the integrity of SKG ileum is compromised in GF, and *P.g*., and *L.m*. monoassociated but not ASF gnotobiotic mice. Curdlan-induced goblet cell loss and ileitis occur after *P.g*. or *L.m*. monoassociation, even though *L.m*. has greater capacity than *P.g*. to stimulate low levels of *Il23a*, *Grp78*, *Tjp1*, mucin, and ZO-1 in naive mice. In contrast, the ASF miniconsortium promotes ileal regulation and barrier integrity with intact goblet cells and ZO-1; induction of *Il10*, *Il6*, and *Ocln*; and no induction of *Il23a,* *Cxcl2*, or ER stress.

### Curdlan induces IL-23–dependent bacterial translocation and uptake by neutrophils and macrophages infiltrating intestinal villi.

**Figure 6:**
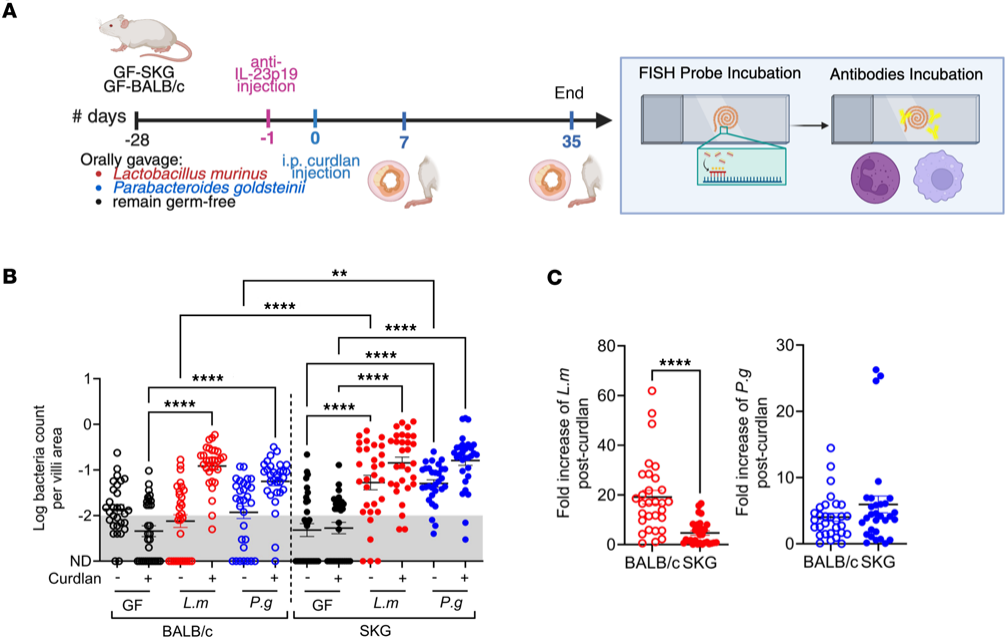
Curdlan promotes *P*. *goldsteinii* and *L*. *murinus* penetration through the ileal mucosa. GF-SKG and GF-BALB/c mice remained GF or were monocolonized with *P.g.* or *L.m.* 4 weeks prior to curdlan i.p. at day 0. Some mice were treated with anti–IL-23p19 at day –1. At 1 week after curdlan, ileum tissues were imaged for bacterial translocation from lumen to villi using FISH with EUB338 probe on Carnoy-fixed tissues. (**A**) Experiment design for Figures 6 and 7. (**B**) Log of the count of bacteria per villi area at week 1 after curdlan. Data show mean ± SEM of the log values with each data point representing 1 ROI. (**C**) Ratio of *L.m*. or *P.g*. bacteria counts in BALB/c and SKG mice treated with or without curdlan. Data show mean ± SEM of the log values, with each data point representing 1 ROI. Two-way ANOVA (**B**) and 1-tailed *t* test (**C**) with **P < 0.01 and ****P < 0.0001.

Since the gut barrier integrity is compromised in SKG but not BALB/c mice and since *P.g*. and *L.m*. act as pathobionts after curdlan, we imaged the physical interaction between the villi and bacteria, staining with the universal bacterial DNA probe EUB338 fluorescence in situ hybridization (FISH) (Figure 6A). In initial experiments, we compared H&E and FISH staining in BALB/c and SKG control mice raised in SPF conditions before and at days 1, 2, 4, and 7 after curdlan (Supplemental Figure 6). In BALB/c ileum, small numbers of bacteria penetrated the lamina propria of the villi at all time points, while in SKG ileum the bacterial signals increased in the villi from day 4 to 7, in the absence of overt inflammation in H&E sections (Supplemental Figure 6). In GF mice, as well as *L.m*. and *P.g*. gnotobiotic mice, we quantified FISH signal in the ileal villi (Figure 6, B and C). *L.m*. and *P.g.* significantly infiltrated the villi of naive SKG, but not BALB/c mice compared with GF ileal villi (Figure 6B). At 7 days after curdlan, *L.m*. and *P.g*. infiltration of the villi significantly increased compared with GF villi in SKG and BALB/c mice (Figure 6B). To measure how curdlan affects the bacterial penetration of the villi, we calculated the fold increase of each bacterium count per villi before and after curdlan in SKG and in BALB/c. Although curdlan did not change the fold increase of infiltration of *P.g*. in the villi, the fold increase infiltration of *L.m*. was significantly reduced in SKG villi compared with BALB/c villi (Figure 6C). This is consistent with the observation that *L.m*. growth is restricted by IL-23p19 after curdlan (Supplemental Figure 3). Together, these data indicate that, in SKG mice monoassociated with *P.g*. or *L.m.*, in which the epithelial barrier is defective, bacteria constitutively infiltrate the lamina propria of the ileal villi. After curdlan, *P.g*. and *L.m*. infiltrate the villi in both BALB/c and SKG ileum, but only in SKG mice is this associated with disease development.

**Figure 7:**
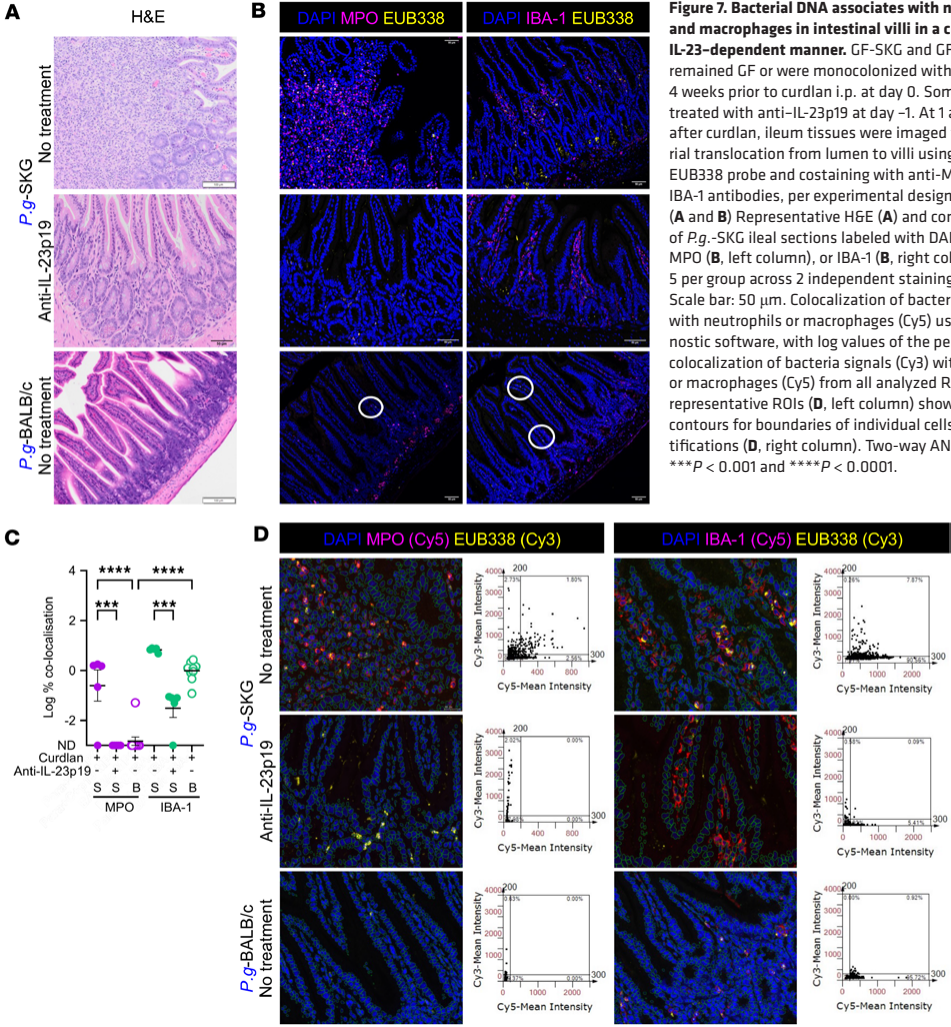
Bacterial DNA associates with neutrophils and macrophages in intestinal villi in a curdlan- and IL-23–dependent manner. GF-SKG and GF-BALB/c mice remained GF or were monocolonized with *P.g.* or *L.m.* 4 weeks prior to curdlan i.p. at day 0. Some mice were treated with anti–IL-23p19 at day –1. At 1 and 5 weeks after curdlan, ileum tissues were imaged for bacterial translocation from lumen to villi using FISH with EUB338 probe and costaining with anti-MPO or anti–IBA-1 antibodies, per experimental design in Figure 6A. (**A** and **B**) Representative H&E (**A**) and confocal images of *P.g*.-SKG ileal sections labeled with DAPI, EUB338, MPO (**B**, left column), or IBA-1 (**B**, right column), with *n* = 5 per group across 2 independent staining experiments. Scale bar: 50 μm. Colocalization of bacteria signals (Cy3) with neutrophils or macrophages (Cy5) using TissueGnostic software, with log values of the percentage of colocalization of bacteria signals (Cy3) with neutrophils or macrophages (Cy5) from all analyzed ROIs in **C**, and representative ROIs (**D**, left column) showing the green contours for boundaries of individual cells and its quantifications (**D**, right column). Two-way ANOVA (**C**) with ***P < 0.001 and ****P < 0.0001.

To understand the mechanism of disease development or protection in SKG relative to BALB/c mice, we next determined the fate of translocated bacterial DNA after entering the villi after curdlan. We stained *P.g*.- and *L.m*.-SKG, GF-SKG, and *P.g.*-BALB/c ileal sections at 1 or 5 weeks after curdlan with EUB338 bacterial DNA probe, antibodies against myeloperoxidase (MPO) expressed by neutrophils, and IBA-1 expressed by macrophages. At 1-week after curdlan, in *P.g*.- or *L.m*.-SKG (Supplemental Figure 7A) but not GF-SKG ileum (Supplemental Figure 8A), EUB338 colocalized with neutrophils and macrophages. This was more intense in *P.g.-* and *L.m*.-SKG at 5 weeks after curdlan (Figure 7, A–C, and Supplemental Figure 7, B and C). In contrast, in *P.g*.-BALB/c mice at 5 weeks after curdlan, neutrophils were absent and bacterial DNA colocalized with resident IBA-1^+^ macrophages (Figure 7, A–C).

With anti–IL-23p19 treatment of *P.g.*-SKG or *L.m.*-SKG mice, MPO^+^ neutrophils were not observed (Figure 7, A–D), but small numbers of resident IBA-1^+^ macrophages remained and colocalized with bacterial DNA in *P.g*.-SKG ileum (Figure 7, A–D). These data demonstrate that bacterial DNA is associated with neutrophils and macrophages in curdlan-treated *P.g.-* and *L.m.*-SKG mice developing ileitis, and with macrophages in BALB/c and IL-23p19–treated SKG mice, suggesting that myeloid cells taking up bacterial DNA may influence persistence of inflammation or tolerance.

### IL-23–dependent bacterial and myeloid cell infiltration of inflamed joint tissues of curdlan-treated SKG mice.

**Figure 8:**
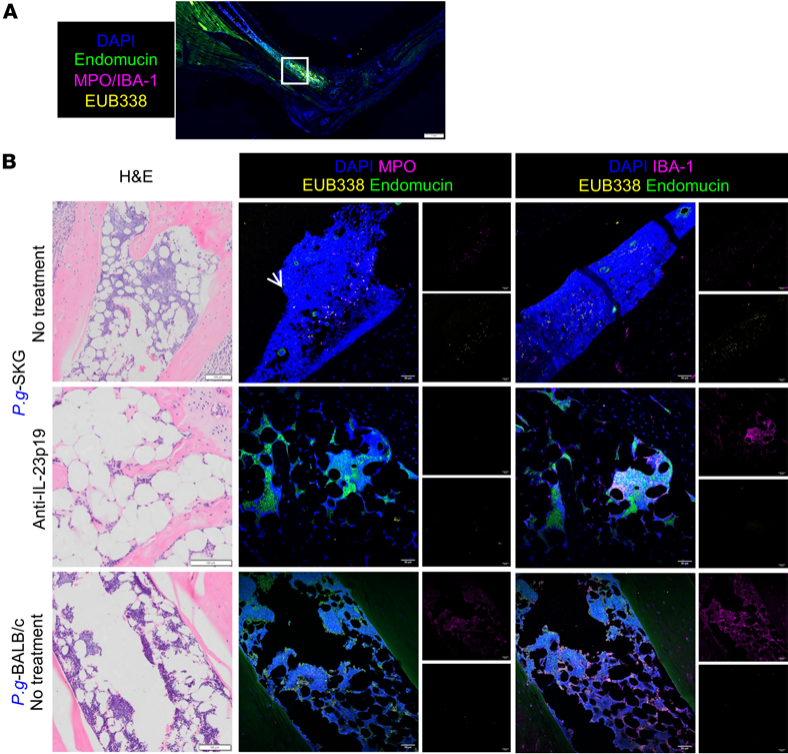
*P*. *goldsteinii* translocation to the bone marrow of inflamed ankles is reduced by anti–IL-23p19 treatment. GF-SKG mice were orally monocolonized with *P.g*. 4 weeks prior to curdlan i.p. at day 0. Some SKG mice were treated with anti–IL-23p19 at day –1. Rear ankle tissues were imaged for the presence of bacteria by FISH staining with EUB338 and anti-MPO or anti–IBA-1 at 5 weeks after curdlan, with *n* = 4 per group across 2 independent staining experiments. (**A** and **B**) Representative fluorescence scanning image of the joint architecture (**A**), with a white outline indicating the imaged area in **B**. Representative H&E and corresponding fluorescence images from bone marrow (**B**), with DAPI (blue), EUB338 (yellow), MPO/IBA-1 (magenta), and endomucin (green). Arrows point to areas of colocalization. Scale bars: 100 μm (**A**) and 50 μm (**B**).

**Figure 9:**
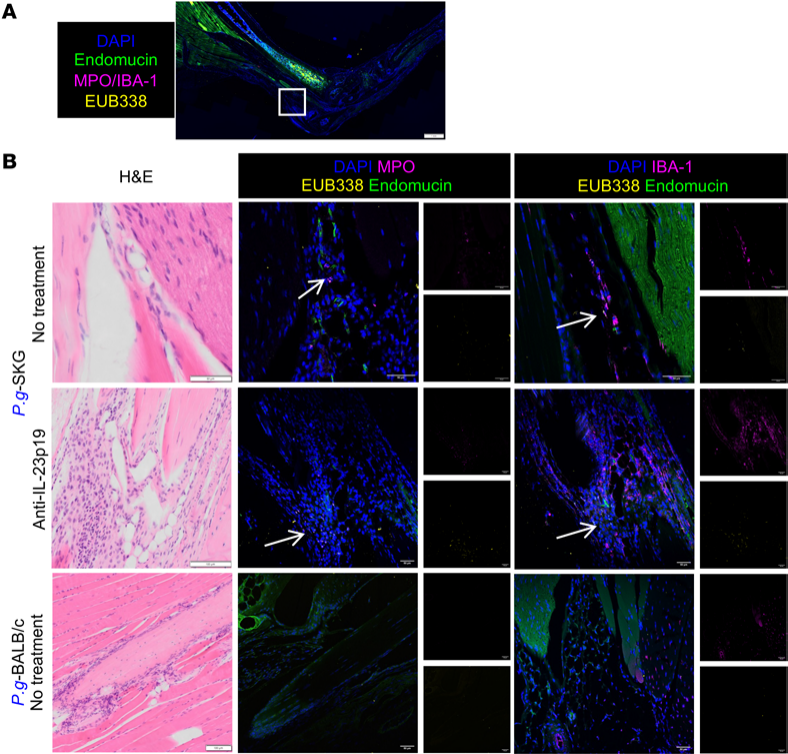
*P*. *goldsteinii* translocation to the enthesis of inflamed ankles is not reduced by anti–IL-23p19 treatment. GF SKG mice were orally monocolonized with *P.g*. 4 weeks prior to curdlan i.p. at day 0. Some SKG mice were treated with anti–IL-23p19 at day –1. Rear ankle tissues were imaged for the presence of bacteria by FISH staining with EUB338 and anti-MPO or anti–IBA-1 at 5 weeks after curdlan, with *n* = 4 per group across 2 independent staining experiments. (**A** and **B**) Representative fluorescence scanning image of the joint architecture (**A**), with a white outline indicating the imaged area in **B**. Representative H&E and corresponding fluorescence images from the enthesis (**B**), with DAPI (blue), EUB338 (yellow), MPO/IBA-1 (magenta), and endomucin (green). Arrows point to areas of colocalization. Scale bars: 100 μm (**A**) and 50 μm (**B**).

To determine whether bacterial DNA reaches the joints, we focused on highly inflamed ankle joint tissues in *P.g*.-SKG mice, without or with anti–IL-23p19, and compared them with GF-SKG and *P.g.-*BALB/c ankle joint tissues at 5 weeks after curdlan, colocalizing EUB338 with MPO and IBA-1 staining (Figures 8 and 9). Bacterial DNA colocalized with MPO^+^ neutrophils and IBA-1^+^ macrophages in the bone marrow (Figure 8, A and B) and enthesis adjacent to joints (Figure 9, A and B) of *P.g*.-SKG but not *P.g*.-BALB/c (Figures 8 and 9) or GF-SKG mice (Supplemental Figure 8, B and C). Bacterial DNA was not found in bone marrow after anti–IL-23p19 (Figure 8), but residual signals remained in the inflamed enthesis colocalizing with both neutrophils and macrophages (Figure 9). After aseptic extraction, no bacteria grew in culture from joint tissues or blood, indicating that the bacterial DNA identified in the inflamed tissues is not associated with viable bacteria (Supplemental Figure 9). These data suggest that bacterial DNA is transported hematogenously to the bone marrow and entheses of inflamed joints by neutrophils and inflammatory macrophages in arthritic *P.g*.-SKG mice, but not in tolerant BALB/c mice. Furthermore, bacterial DNA transported by myeloid cells to the bone marrow but not the enthesis is IL-23p19 dependent.

### Bacterial DNA signals are present in the intestine and synovial tissues in patients with SpA.

**Figure 10:**
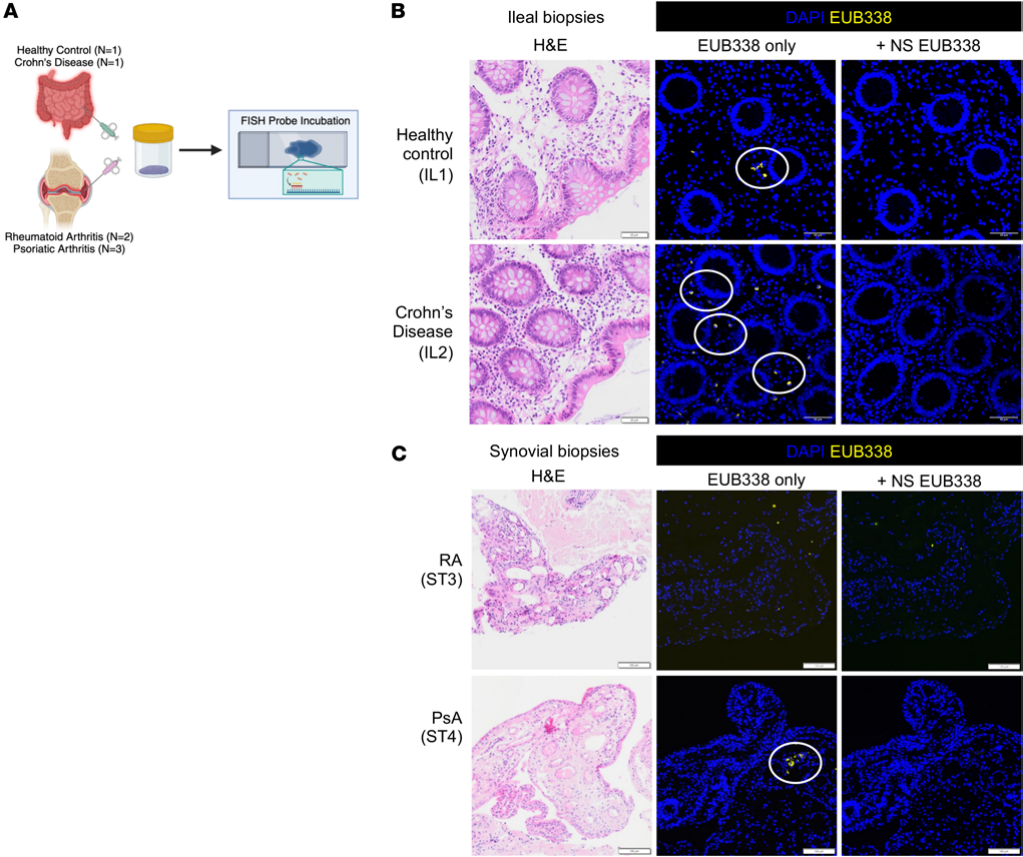
PsA but not RA synovial biopsies contain bacterial DNA. Three PsA and 2 RA synovial biopsies were assessed for the presence of bacteria by imaging using FISH staining with EUB338. Ileal biopsies from 1 healthy individual and 1 individual with Crohn’s disease were used as positive controls (Table 1). Nonsense EUB338 probes were added to verify the specific EUB338 signals. (**A**) Graphical representation of experiment design. (**B** and **C**) Representative fluorescence images by confocal microscopy of ileal biopsies IL1 and IL2 (**B**) and synovial biopsies ST3 and ST4 (**C**) with DAPI (blue) and EUB338 (yellow). Circles indicate areas of EUB338^+^ signals. Scale bars: 50 μm (**B**) and 100 μm (**C**).

Patients with PsA may develop synovitis of large and small joints, enthesitis and bone marrow edema, and have an increased risk of IBD, whereas patients with rheumatoid arthritis (RA) develop small to large joint synovitis but not with associated IBD (28, 29). To determine whether bacterial DNA might also reach joints in PsA or RA, we stained positive control Crohn’s IBD ileal sections and synovial tissue sections from patients with PsA or RA, with FISH (Figure 10A). EUB338 signals were apparent in Crohn’s ileitis (Figure 10B) and in 1 of 3 PsA and 0 of 2 RA synovial biopsies (Figure 10C). These data provide preliminary evidence of bacterial DNA translocation to human SpA joints.

### Myeloid cells from SPF-SKG mice but not SPF-BALB/c or GF-SKG mice are arthritogenic.

**Figure 11:**
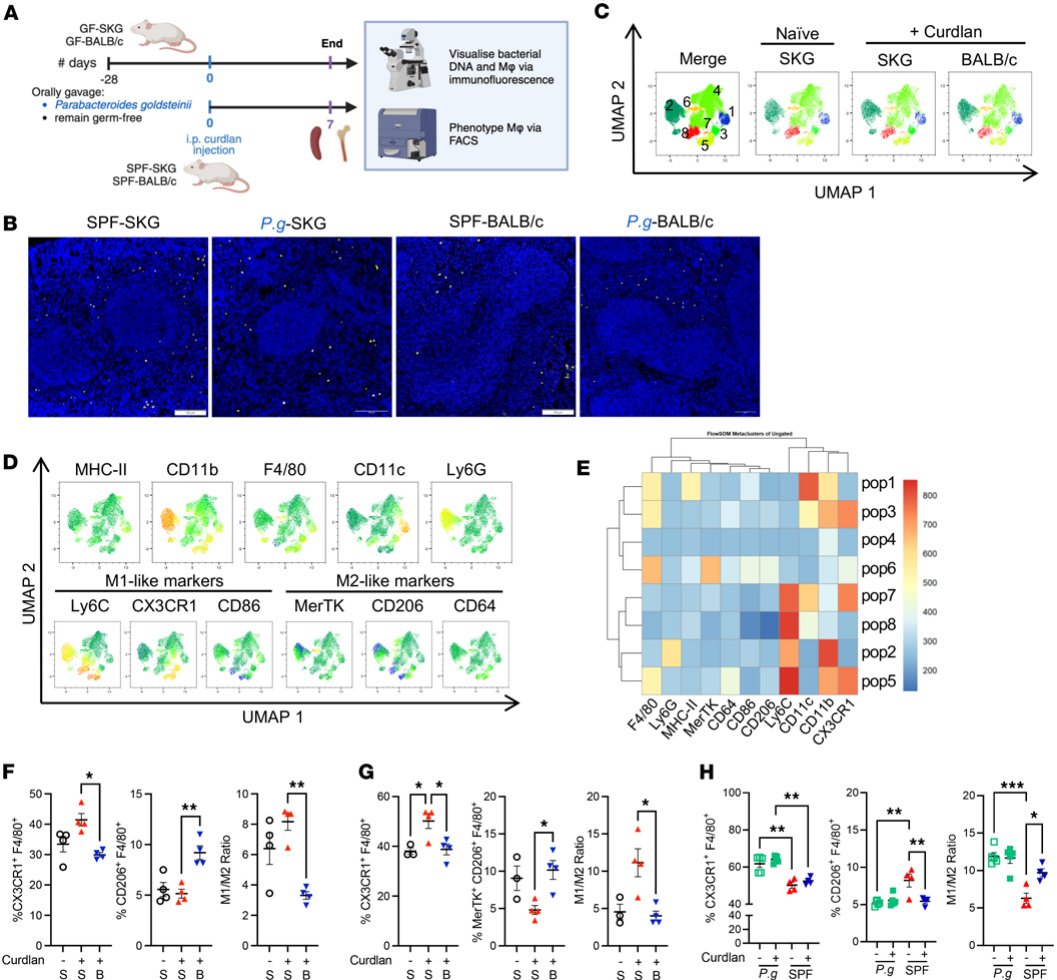
Myeloid cells in the spleen and bone marrow of SKG mice are proinflammatory. (**A**–**H**) Experiment design of macrophage phenotyping experiments (**A**) related to **B**–**H**. (**B**) Representative fluorescence images by confocal microscopy of EUB338^+^ signals in the spleen sections from the indicated groups. See also Supplemental Figure 10. Single-cell suspensions from the spleen and bone marrow were analyzed by FACS. (**C**–**E**) Unsupervized clustering analysis (**C** individual marker expression displayed on UMAP (**D**), and heatmap data (**E**). (**F** and **G**) Percentage of CX3CR1^+^F4/80^+^, MerTK^+^CD206^+^F4/80^+^ manually gated in live CD45.2^+^TCRβ^–^CD19^–^CD11b^+^Ly6G^–^ cells, and M1/M2 ratio in the spleen (**F**) and bone marrow (**G**). (**H**) M1-like, M2-like and M1/M2 ratio from naive and 1-week curdlan-treated *P.g*.-SKG compared with SPF-SKG gated as in **F**. *n* = 4–5 in 2 experiments. Data show mean ± SEM, with each data point representing an individual mouse. One-way ANOVA (**F**, **G**, **J**, and **K**) and 2-way ANOVA (**H**) with *P < 0.5, **P < 0.01, ***P < 0.001.

To understand whether DNA is also transported hematogenously beyond the resident macrophages of the gut in curdlan-treated BALB/c mice, we compared spleens of naive and curdlan-treated SKG and BALB/c mice raised under SPF or *P.g.-*monoassociation conditions (Figure 11A). In all groups of SKG and BALB/c mice tested, without or with curdlan treatment, EUB338 signals are localized to the red pulp of the spleen (Figure 11B). These signals are located in the extrafollicular regions (Supplemental Figure 10, A and B), where F4/80^+^ macrophages were present (Supplemental Figure 10C), suggesting constitutive hematogenous transport of bacterial DNA associated with macrophages from the intestine to the spleen.

Since macrophages transported bacterial DNA from the intestine of *P.g*.-SKG mice to bone marrow and spleen after curdlan, and from intestine to spleen in BALB/c mice, we reasoned that different macrophage subtypes participating in bacterial clearance may contribute to tolerance or arthritis development. To assess this, we first compared by flow cytometry the phenotype of macrophages in spleen and bone marrow of naive SKG, 7 days postcurdlan SKG, and 7 days postcurdlan BALB/c mice housed in SPF conditions (Figure 11A). Unsupervized clustering identified 2 of the 8 populations to be differentially expressed: population 5 expressing inflammatory M1-like marker CX3CR1, and population 6 expressing tolerant M2-like markers, MerTK and CD206 (Figure 11, C–E). Using manual gating, inflammatory M1-like macrophages were present in higher proportion in the spleen (Figure 11F) and bone marrow (Figure 11G) of curdlan-treated SKG compared with naive SKG or curdlan-treated BALB/c mice, while M2-like macrophages were enriched in curdlan-treated BALB/c compared with SKG mice. The ratios of M1-like proinflammatory macrophage over M2-like tolerant macrophages were higher in curdlan-treated SKG mice relative to naive SKG and curdlan-treated BALB/c in the spleen (Figure 11F) and bone marrow (Figure 11G). *P.g.-*SKG and GF-SKG spleens were intrinsically enriched in M1-like and devoid of M2-like macrophages compared with SPF spleens (Figure 11H and Supplemental Figure 11A). Thus, colocalization of neutrophils and macrophages with bacterial DNA after curdlan in the gut is associated with M1 macrophage enrichment in spleen and bone marrow in SKG mice.

**Figure 12:**
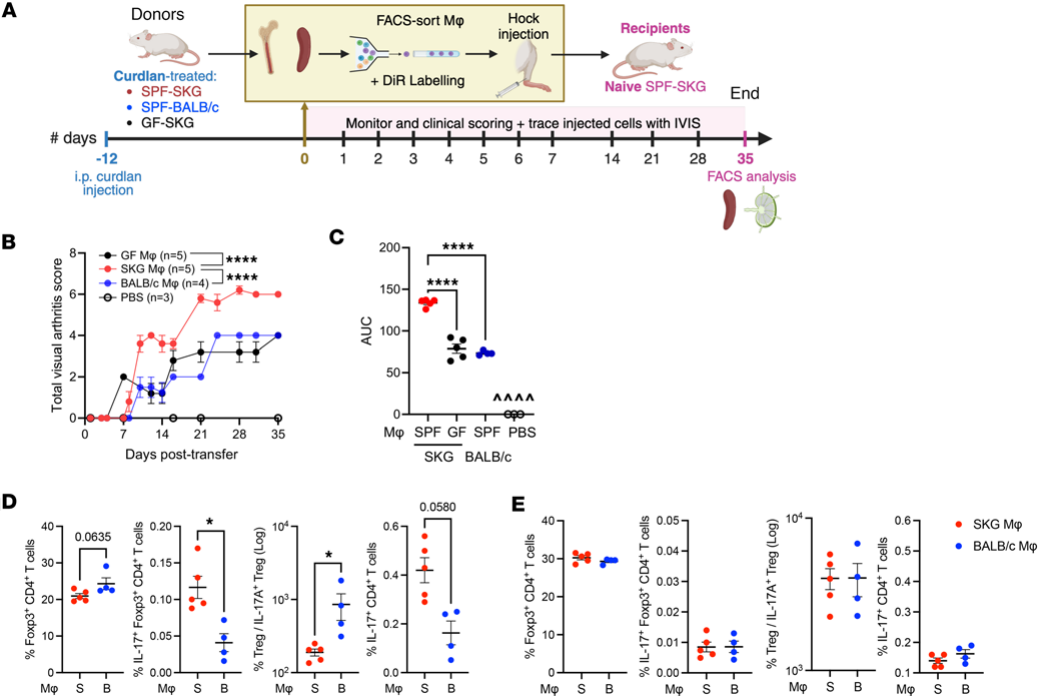
SKG-derived proinflammatory myeloid cells are arthritogenic. (**A**–**E**) Experiment design of macrophage adoptive transfer experiments (**A**) related to **B**–**E**. CD45.2^+^TCRβ^–^CD19^–^CD11b^+^Ly6G^–^ myeloid cells were sorted from the spleen and BM of SPF-SKG, SPF-BALB/c, or GF SKG and injected into naive SPF-SKG mice at day 0 (s.c. hock) and compared with mock injected mice with PBS. (**B** and **C**) Visual arthritis score (**B**) and AUC (**C**) are shown. (**D** and **E**) At 35 days after injection end-point, Tregs and conventional T cells from the popliteal lymph nodes (**D**) and spleen (**E**) were analyzed by FACS. *n* = 4–5 in 2 experiments. Data show mean ± SEM, with each data point representing an individual mouse. One-way ANOVA (**B** and **C**) and Mann-Whitney *U* test (**D** and **E**) with *P < 0.5 and ****P < 0.0001.

To demonstrate the potential pathogenicity of these macrophage subsets, we sorted CD45^+^CD11b^+^F4/80^+^Ly6G^–^ macrophages by FACS from spleen and bone marrow of curdlan-treated SPF-SKG, SPF-BALB/c (proportions of which carry bacterial DNA), or GF-SKG mice, which are M1-skewed but carry no bacterial DNA. These cells were adoptively transferred s.c. in the hock of naive SKG recipients (Figure 12A). Sham-injected SKG mice received PBS. SPF-SKG macrophages induced severe arthritis within 21 days, while SPF-BALB/c macrophages or GF-SKG macrophages both induced a background level of mild arthritis and transient weight loss (Figure 12, B and C, and Supplemental Figure 11B). Thus, the combined effects of bacterial DNA and M1 skew amplify the arthritogenicity of SPF-SKG macrophages.

Although DiR-labeled adoptively transferred macrophages were only detectable at the injection site up to day 21 (Supplemental Figure 11, E–H), IL-17A^+^CD4^+^Foxp3^+^ Tregs and conventional T cells expanded in the popliteal joint draining lymph nodes (dLN) (Figure 12D and Supplemental Figure 11, C and D) and not in the spleen of mice receiving SPF-SKG macrophages (Figure 12E). In mice receiving SPF-BALB/c macrophages, the ratio of total Foxp3^+^ Tregs to IL-17^+^ Tregs was higher (Figure 12D). These data indicate that proinflammatory SPF-SKG M1-like macrophages promote expansion of IL-17A^+^ autoreactive conventional and Tregs and arthritis, while SPF-BALB/c M2-like macrophages regulate IL-17^+^ Treg and arthritis severity in naive SKG recipients.

### CD4^+^ T cell proliferation in the spleen expands to the joints in SKG mice after curdlan.

**Figure 13:**
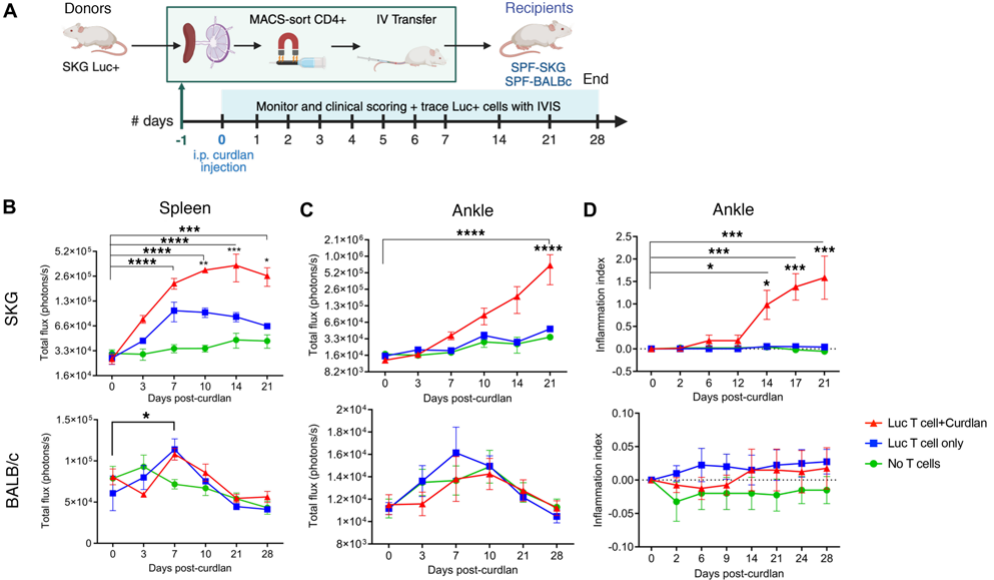
Tracking CD4^+^ T cells during early development of curdlan-induced SpA in SPF-SKG mice. SPF-SKG and SPF-BALB/c mice were injected with 5 × 10^6^ SKG.luc^+^CD4^+^ T cells 1 day prior to curdlan, imaged via IVIS, and scored for visual arthritis, with *n* = 6 per group across 2 independent experiments. (**A**) Experimental design. (**B** and **C**) Total bioluminescence flux (photons/s) in the spleen (**B**) and rear ankles (**C**). (**D**) Clinical arthritis scores in the rear ankles. Data show mean ± SEM. Statistical analysis: Ordinary 2-way ANOVA (Tukey’s multiple comparison) test with *P < 0.5, ***P < 0.001, ****P < 0.0001.

To confirm the primary sites of T cell proliferation in response to antigen presentation after curdlan, we adoptively transferred SPF-SKG or SPF-BALB/c mice with 5 × 10^6^ SKG.luc^+^CD4^+^ T cells with or without curdlan. We then monitored the development of arthritis and the fate of transferred T cells by in vivo bioluminescence imaging over time (Figure 13A).

**Figure 14:**
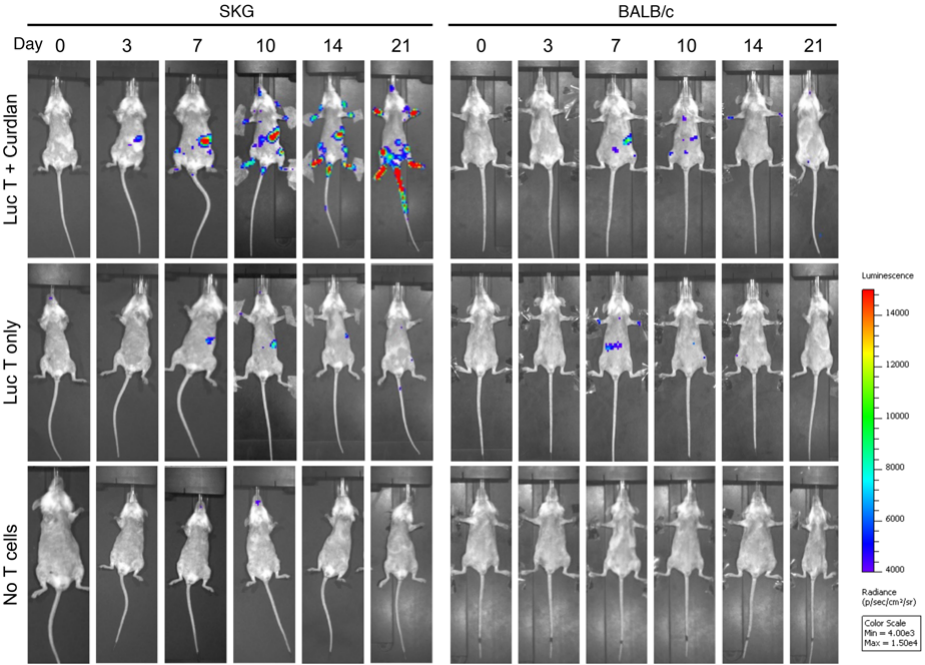
Tracking CD4^+^ T cells during early development of curdlan-induced SpA in SPF-SKG mice. SPF-SKG and SPF-BALB/c mice were injected with 5 × 10^6^ SKG.luc^+^ CD4^+^ T cells 1 day prior to curdlan, imaged via IVIS and scored for visual arthritis, with *n* = 6 per group across 2 independent experiments, per experimental design in Figure 13A. Representative ventral bioluminescence images in SKG and for BALB/c recipients.

SKG.luc^+^CD4^+^ T cell bioluminescence signal initially increased significantly in the spleen of recipient mice within 7 days, without or with curdlan (Figure 13B and Figure 14). Continued T cell expansion in spleen as well as T cell recruitment and expansion in large joints occurred only in SKG mice receiving curdlan (Figure 13C and Figure 14). This preceded the initiation of arthritis in the joint from day 14 after curdlan (Figure 13D). In contrast, SKG.luc^+^CD4^+^ T cell bioluminescence signal increased transiently in the spleen of BALB/c mice at day 7 and did not progress to joint recruitment or arthritis (Figure 13, B–D, and Figure 14). These data suggest that antigen presentation in the SKG spleen, where M1 macrophages predominate, drives autoreactive SKG CD4^+^ T cell expansion. With systemic curdlan and only in an SKG host, they acquire migratory capacity to the joints. In contrast in BALB/c hosts, M2 macrophage-dominant antigen presentation regulates SKG CD4^+^ T cell expansion and joint migratory potential, controlling development of SpA.

## Discussion

In patients with SpA, gut inflammation is linked to chronic spondyloarthritis (1, 30). It is hypothesized that loss of tolerance to gut commensal bacteria and innate immune exposure to entero-invasive species is followed by self-/bacterial peptide presentation, particularly in the context of HLA-B27 (31). How and where this might happen are unclear. In SpA-susceptible ZAP-70^W163C^ SKG mice, Gram-negative dysbiosis is associated with disease severity (14, 15). Other features of SKG T cells that predispose to defective tolerance include deficiencies of positive and negative thymic selection, Treg function, CD4^+^CD8^+^ double-positive intraepithelial cytotoxic T cells, and control of the response to MMTV superantigen (32). In this study, we dissected the mechanisms underlying curdlan-induced myeloid cell exposure to known commensals *P.g*. or *L.m*. in gnotobiotic SKG hosts that developed ileitis and arthritis, ASF-gnotobiotic SKG that developed arthritis, and tolerant BALB/c mice. Features of disease susceptibility in *P.g.*- or *L.m.*-SKG mice included ileal expression of *Il23a* and ER stress genes, ileal barrier dysfunction, and goblet cell loss, as well as uptake of bacterial DNA within the lamina propria by neutrophils and inflammatory M1 macrophages and the transport of bacterial DNA to spleen, peri-articular bone marrow, and entheses. In contrast, tolerant BALB/c mice exposed to the same bacteria and curdlan maintained goblet cells and epithelial tight junctions and expressed low levels of *Il23a*. *P.g*. and *L.m*. translocated to BALB/c lamina propria after curdlan but, in the absence of inflammation, were taken up by intestinal resident macrophages, with transport to spleen but not to the joint. ASF-SKG mice also actively regulated the ileal barrier. Importantly, curdlan-treated SPF-SKG spleen/bone marrow macrophages were sufficient to transfer severe arthritis to naive SKG recipients, associated with Th17 expansion, while BALB/c or GF-SKG macrophages transferred only mild arthritis associated with Th17 regulation. Bacterial DNA was also identified in synovial tissue of a PsA patient with axial involvement, suggesting similar translocation from peripheral sites to joint in patients with SpA.

Previously, DNA from mucosal bacteria, such as *Prevotella spp.*, was similarly found in spleen, liver, lung, serum, mesenteric LN, eyes, and ankle joints in HLA-B27 transgenic and control rats with or without SpA, but its significance was unclear (33, 34). In SKG and BALB/c gnotobiotic mice, inflammatory outcomes in the ileum, joint, and enthesis after curdlan varied according to host mouse strain, gut bacterial species, and treatment with anti–IL-23p19. While the direct mechanism of macrophage activation by bacterial DNA in SKG mice is not yet known, it is interesting to note that DNA-loaded membrane vesicles from gut microbiota circulated and, by evading host nucleases, activated type 1 IFN via cGAS/STING in remote host macrophages. This innate training in turn promoted protection against host viral infection (35). On the other hand, MerTK^+^CD206^+^ M2-like macrophages associated with bacterial DNA were predominant in the gut of BALB/c mice and of SKG mice treated with anti–IL-23p19, reinforcing intestinal tolerance to bacteria. M2-like tissue-resident macrophages in the splenic red pulp are similarly positioned to capture blood-borne antigens, regulate adaptive immune responses, and maintain tolerance (36, 37). It will be of interest to determine whether dysbiotic and homeostatic-derived membrane vesicles can reinforce macrophage polarization.

In contrast, ileitis was severe only in curdlan-treated *L.m.-* and *P.g.*-SKG mice. In the absence of bacteria, SKG GF mice were primed to express ileal *Il23a* after curdlan, but ER stress gene upregulation and loss of goblet cells only occurred with curdlan and *L.m*. or *P.g*. colonization. *L.m*. and *P.g*. infiltrated intestinal villi of SKG mice associated with variable low-level expression of *Il23a*, *Il6*, *Cxcl2*, and *Grp78* even before curdlan. Thus, the SKG-GF monocolonized ileal environment is poised for stress-associated loss of ileal integrity and recruitment of neutrophils and inflammatory macrophages after curdlan. Similar features of damaged intestinal mucosal barrier, bacterial adherence to gut villi, and invasion of the lamina propria and myeloid cell infiltration were also described in patients with AS (26), and we observed bacterial DNA in the lamina propria of patients with Crohn’s ileitis.

Intriguingly, susceptibility to ileitis in SKG mice could be mitigated by the ASF miniconsortium, which regulated *Il23a* and ER stress and upregulated *Il10*, preserving goblet cells and ZO-1^+^ tight junctions. The capacity of certain commensals to regulate ileal susceptibility is consistent with previous evidence that fecal material or fecal *Ruminococcus gnavus* isolates from patients with RA or AS, but not healthy control fecal material, aggravated the development of zymosan or curdlan-induced arthritis in SKG mice (38, 39). Future studies of the precise regulatory mechanisms involved, such as NF-κB inhibition or ILC3 expansion (13, 40) and why these were insufficient to mitigate arthritis in ASF-SKG mice, will be important for development of precision probiotics or other ileal immunomodulators in SpA.

Although neutrophils and inflammatory macrophages colocalized with *P.g*. or *L.m*. DNA, which resulted in SpA in SKG gnotobiotic mice, *P.g*. and *L.m*. have been shown to act as probiotic or regulatory commensals in other contexts and genetic backgrounds (17–24). Specifically in the gut, *P.g*. and *L*. *reuteri* induced the differentiation of CD4^+^CD8αα^+^ intraepithelial lymphocytes (CD4-IELs) (41–43), a class of innate-like T cells that contribute to intestinal tolerance and bacterial responses through microbiota-immune system interactions. As patients with AS and SKG mice have reduced CD4-IELs associated with poor mucosal immunosurveillance (10, 15), the probiotic potential and protective tolerance mechanisms normally induced by *P.g*. and *L.m*. are reduced. In the hostile, IL-23–rich SKG gut microenvironment, *L.m*. expansion was limited. *Prevotella* strains have also been shown to metagenomically adapt with arthritogenic virulence factors (44), rendering them more pathogenic in dysbiotic environments.

IL-23 is canonical in SpA pathogenesis (45, 46). In gnotobiotic SKG mice, inflammatory macrophages and neutrophils carried gut bacterial DNA to the joint-associated myeloblastic bone marrow and entheses. Remarkably, anti–IL-23p19 suppressed retention of bacterial DNA in bone marrow but not inflamed entheses. Bone marrow edema is a characteristic radiological feature of human AS and PsA (47), and the enrichment of IL-23–secreting CD14^+^ myeloid cells in SpA patient bone marrow likely contributes (48). Inflammatory macrophages and neutrophils are similarly enriched in the entheses, synovium, and gut of patients with SpA, associated with disease severity. IL-23 inhibitors are effective in PsA but not for the poly-enthesitis of the spine in AS (49, 50). While it is difficult to obtain periarticular bone marrow or entheseal tissue from patients with PsA or AS, we observed bacterial DNA in a perivascular location in synovial tissue from a patient with PsA with axial involvement — i.e., SpA-like disease. It will be of interest to determine whether bacterial DNA is also retained in entheses from patients with AS, promoting IL-23–independent inflammation and/or T cell activation (51).

In SKG mice, inflammatory M1-like macrophages expressed CX3CR1, CD11b, and Ly6C. Similar CX3CR1^+^ inflammatory macrophages in patients with AS express CD59, IL-23, and α4β7, suggesting their intestinal origin (52). Consistent with the development of an immune response to self-antigens and gut pathobionts in HLA-B27^+^ patients with AS, clonally expanded T cells in the blood and synovium were shown to recognise self and bacterial antigens (53, 54), supporting an antigen-presenting role of CX3CR1^+^ inflammatory macrophages. CX3CR1^+^ inflammatory macrophages and their bacterial DNA cargo may persist in SpA for several reasons, including a lack of cytotoxic T cells or innate lymphoid cells (10) and resistance to complement-mediated lysis, which contributes to the avoidance of sterilizing immunity (55). Several pieces of evidence support a collaborative relationship between inflammatory CX3CR1^+^ macrophages and neutrophils. At the onset of antigen-induced arthritis, CD68^+^ macrophages recruited neutrophils into the synovial lining niche through CXCL1/CXCR2 chemokines and vascular E-selectin (56). Adoptive transfer of MIF^+^ neutrophils expanded inflammatory macrophages in joints of SKG mice (57). Clodronate liposomes that suppress the function of neutrophils while depleting a subset of macrophages (58) also blocked development of 3 models of bacteria*-*induced arthritis (58–61). The current studies demonstrate that this collaboration originates in the uptake and transport of invasive gut bacterial DNA.

The present study has limitations. The intestinal immune system develops differently in GF and conventional SPF mice, as exemplified by the skewed M1 phenotype of GF splenic macrophages that we observed. Furthermore, our experimental model was limited to gavage of adult GF mice rather than maternal microbial transmission. In addition, we were technically limited to visualizing bacteria using a universal FISH probe in a small number of synovial biopsies, and the human tissue fixative precluded staining with additional immunofluorescent antibodies. Nonetheless, GF and gnotobiotic SKG mice clarify the mechanistic role played by intestinal bacteria in SpA.

In conclusion, after curdlan, proinflammatory M1 macrophages containing DNA from gut bacteria drive Th17 differentiation and IL-23–dependent ileitis and arthritis in susceptible ZAP70^W163C^ SKG hosts. In SpA-resistant BALB/c hosts, bacterial DNA-associated M2 macrophages maintain T cell regulation. Our findings suggest new opportunities to modulate disease susceptibility through the gut-bacterial interface and myeloid antigen-presenting cells.

## Methods

Supplemental Methods and Supplemental Table 1 are available online with this article.

### Sex as a biological variable.

Our study exclusively examined female mice because the disease phenotype is dominant in females. For studies in patients, both men and women were included.

### Study approval.

Approval for all animal experiments was obtained from the University of Queensland animal ethics committee. Patient ileal biopsies samples were collected at Mater Hospital (Brisbane, Queensland, Australia) in accordance with the recommendations of the Mater Health Services Human Research Ethics Committee (HREC/14/MHS/125 and HREC/MML/92379) for the Mater Inflammatory Bowel Disease Biobank. Arthroscopic synovial tissue samples were collected at Flinders Medical Centre (Adelaide, South Australia, Australia) under the approval of Southern Adelaide Local Health Network (SALHN) Human Research Ethics Committee (SALHN/HREC/396.10).

### Statistics.

GraphPad Prism software was used for statistical analysis. The data in the figures are presented as the mean ± SEM. For data with 2 groups, 1- or 2-tailed *t* tests were used. For data with more than 2 groups, Mann-Whitney *U* tests or one- or two-way ANOVA were used. *P* < 0.05 was considered statistically significant.

### Data availability.

Values for all data points in the figures are provided in the Supporting Data Values file.

## Author contributions

RT and ASB acquired funding. RT, ASB, and LMR conceived and designed the study. ASB, BC, RG, AJC, MAR, AS, CA, and YL performed experiments and analyzed data, under the supervision of ASB, RT, MM, JB, and MDW. BC, ASB, and RT prepared and edited the manuscript. All authors discussed the results, read, commented, and edited on the final manuscript.

## Supplementary Material

Supplemental data

Supporting data values

## Figures and Tables

**Table 1:**
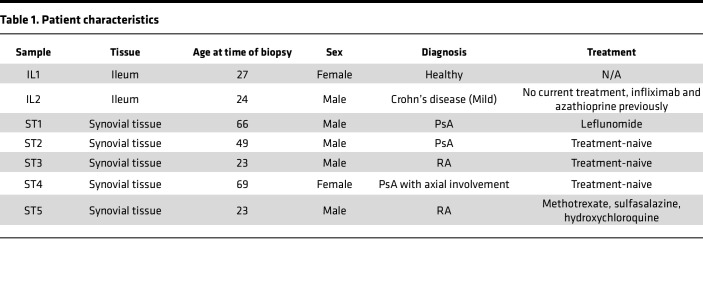
Patient characteristics

## Version 1 07/31/2025

In-Press Preview

## Version 2 09/09/2025

Electronic publication
