# Supplementary material for: Proinflammatory macrophages transporting gut-derived bacterial DNA drive autoimmune arthritis in spondyloarthropathy
Source: JCI Insight. 2025 Jul 31;10(17):e188028. doi: 10.1172/jci.insight.188028 (PMC12487687; doi:10.1172/jci.insight.188028)
Supplement: Supplemental data [file jciinsight-10-188028-s097.pdf]

Supplemental Information

Pro-inflammatory macrophages transporting gut-derived bacterial DNA drive autoimmune arthritis in spondyloarthropathy

Benjamin Cai[1], Rabina Giri[2], Amy J Cameron[1], M Arifur Rahman[1#], Olga Zbarskaya[1], Annabelle Small[3], Christopher Altmann[3], Yenkai Lim[1], Linda M Rehaume[1], Mark Morrison[1], Mihir Wechelakar[3], Jakob Begun[2], Anne-Sophie Bergot[1,4*], Ranjeny Thomas[1,4*]

[1] Frazer Institute, Faculty of Health, Medicine and Behavioural Sciences, The University of Queensland, Brisbane, Australia

[2] Mater Research Institute, The University of Queensland, Brisbane, Australia

[3] College of Medicine & Public Health, Flinders University, Bedford Park, South Australia, Australia

[4] Co-senior authorship

[*]Correspondence: a.bergot@uq.edu.au (A-S.B.), ranjeny.thomas@uq.edu.au (R.T.)

[#]Present address: QIMR Berghofer Medical Research Institute, Brisbane, Australia

-

Supplemental Methods
Supplemental Table 1
Supplemental Figures S1-S11

Supplemental Methods

Mice

Female BALB/c, SKG mice (ZAP-70^{w163c}-mutant BALB/c mice), originally obtained from S. Sakaguchi (University of Kyoto) were bred and housed under SPF or Germ-Free conditions at The University of Queensland Translational Research Institute (TRI) Animal Facility under the guidelines of University of Queensland (UQBR). Mice were used 8-16 weeks of age. For adoptive transfer of T cells, SKG.luc^{+} mice, expressing the luciferase gene under the ubiquitin promoter of β-actin, were generated by backcrossing SKG and BALB/c.luc^{+} derived from luciferase-expressing transgenic B6-L2G8 mice generated by R. Negrin (Stanford University, Stanford, USA), for two generations. The generated SKG.luc^{+} mice were homozygous for the ZAP-70 mutation and heterozygous for the luciferase gene. SKG.luc^{+} offspring were screened for luciferase activity using blood from a retro-orbital bleed in the *in vitro* luciferase assay Steady-Glo (Promega). The inflammation index is the change of width of the ankle, wrist and footpad from baseline over time. Mice were sacrificed at indicated timepoints using CO_2 euthanasia.

Bacterial colonisation of germ-free mice

Groups of mice were orally gavaged with faeces from live donors colonised with *Parabacteroides goldsteinii* (ASF519) or *Lactobacillus murinus* (ASF361) 2 to 4 weeks prior to curdlan injection (15 mg/mL, 200 µL i.p., Wako). Anti-mouse IL-23p19 antibody (60 µg/mouse, i.p., Eli Lilly and Company, Indianapolis, USA) was injected one day prior to curdlan injection. Control groups were injected with control isotype. Mice were weighted and scored for visual signs of arthritis weekly until endpoint.

Bacteria culture and *in vitro* assay

Caco-2 cells were seeded at 5 x 10^4 cells per well on 12 well glass-bottom plate and grown until a formed monolayer. *Lactobacillus murinus* was grown with anaerobically prepared MRS broth and

Parabacteroides goldsteinii with anaerobic BHI broth. Bacteria were used when O.D(600nm) = 0.2. Caco-2 monolayers were infected with either *L. murinus* or *P .goldsteinii* for 6 hours, followed by 3x PBS washes. Cells were then fixed with 4% paraformaldehyde (PFA) for 15 minutes, permeabilised with 0.1% Triton X for 15 minutes, followed by blocking with 5% goat serum for 1 hour. Cells were then stained with anti-ZO-1 antibody (Cell signalling) and counter-stained with DAPI (Sigma-Aldrich) and WGA-FITC (Sigma) before imaging with Olympus FV3000 confocal microscope.

Isolation and analysis of single cells from tissues

Spleen was cut into small pieces with scissors and enzymatically digested with a cocktail of collagenase D (at 2 mg/mL; Roche) and DNase1 (at 10 µg/mL; Sigma-Aldrich) for 30 min at 37 °C. Cell suspension was then passed through 70 µm cell strainers. Cell pellets were then incubated with ACK buffer to lyse erythrocytes. Lymph nodes were mashed and passed through 70 µm cell strainers. They were then centrifuged and used for FACS analysis.

For intraepithelial and lamina propria lymphocytes, small intestines were collected, and Peyer's patches (PP) were excised and mashed through cell strainer to collect cells. Intestines were cut open longitudinally and intestinal luminal contents were washed with cold PBS, then cut into 1 cm pieces. The pieces were stirred at 37 °C for 20 min in RPMI supplemented with 5% FCS, penicillin/streptomycin, 1 mM EDTA and 1 mM DTT. The intraepithelial lymphocytes (IELs) in the supernatant were collected and washed. Residual tissue was cut into fine pieces and stirred in RPMI-1640 supplemented with 10% FCS, 1mg/mL collagenase D (Roche) and 50 µg/mL DNase I (Roche) at 37 °C for 45 min. The lamina propria lymphocytes (LPLs) in the supernatant were collected and washed with RPMI 1640. Epithelial cells were removed using 70% Percoll® (Sigma-Aldrich) for 20 min at 2000 rpm.

For synovial tissues, rear paws were collected in 5% RPMI. Skin was peeled off, toes (at distal phalanges) and tibia (about 0.5cm above talus bone) were cut off, excess muscle was removed,

exposing the tendons and tissues were open between digits. Bone marrow was flushed by placing the ankle toes up in an 500µl tube placed in a 1.5mL Eppendorf tube and spun at 10,000 rpm for 30 sec. Tissues were digested with RPMI-1640 supplemented with 10% FCS, 0.5 mg/mL hyaluronidase (Sigma) and 1mg/mL collagenase D (Roche) for 1 hour at 37°C. Following passing through a 70 µm cell strainer, the cells were centrifuged and used for FACS analysis.

For bacteria culture, rear paws were harvested aseptically within a biosafety cabinet after a 5-minute bath of the whole mouse in 1% Virkon solution. The whole leg was collected and further incubated in 20 µg/mL gentamicin antibiotics (non-cell penetrant) for 1min, then washed with sterile PBS [59]. Skin was peeled off, toes (at distal phalanges) and tibia (about 0.5cm above talus bone) were cut off, excess muscle was removed sterily in a 5cm petri dish. Tissues were crushed in a tissue homogeniser, beads-beaten for 5000x3x60 using Precellys (Bertin), and then immediately placed in growth medium in anaerobia for up to 7 days.

Antibodies and flow cytometry

Single cell suspensions were obtained per above protocols. Cells were stimulated with Cell Activation Cocktail (Biolegend; phorbol myristate acetate and ionomycin), treated with monensin (eBioscience) for 4 hours if staining for intracellular cytokines. Samples were firstly stained with LIVE/DEAD™ Aqua (Invitrogen) or Fixable Viability Stain 440UV (BD) in PBS. Fluorochrome-conjugated monoclonal antibodies for cell-surface markers were stained for 25 minutes at 4℃ per manufacturers' protocols. For intracellular staining, mouse regulatory T cell staining kit (eBioscience) was used for intracellular staining of Foxp3 following manufacturer's instructions, as well as IFNγ (BD) and IL-17A (Biolegend). Fixation and permeabilisation was done at 4℃ for 45 minutes followed by antibodies for intracellular cytokines for 25 minutes in the dark. Flow cytometry data were acquired on a Fortessa X-20 cytometer (BD) and analysed with FlowJo software v9.

Gene expression assay

Ileum samples were collected, snap frozen and stored at -80℃ until mRNA was extracted using QIAGEN RNeasy kit. 500 ng of mRNA were synthesized in cDNA using the Sensifast cDNA synthesis kit as per manufacturer's instruction. qPCR was processed using 100 ng of cDNA as per manufacturer's instruction (SensiFAST SYBR Lo-ROX Kit, Bioline).

Immunohistochemistry, Fluorescent In-Situ Hybridisation and Immunofluorescence

Formalin/Carnoy-fixed paraffin-embedded human ileum, human synovial biopsies, mouse ileum, colon, spleen, and decalcified ankle blocks were cut into 4μm sections, deparaffined with xylene (3 x 5 min), rehydrated with alcohol (100% EtOH 2 x 5 min followed by 80% EtOH 1 x 5 min) and air-dried before staining.

For IHC, H&E staining was performed on 4μm FFPE sections. Goblet cells were labelled using Alcian Blue staining on deparaffined sections of ileum. IHC-labelled slides were scanned with Olympus VS200 Research Slide Scanner.

For FISH, tissue sections were incubated with 2 μM FISH probes diluted in of 0.9M NaCl, 0.02M Tris, 0.01% SDS, and 20% Hi-Di Formamide at 46 °C overnight. Sections were then washed with PBST and blocked with 1% BSA in PBS containing 0.2% Triton X-100 and 0.05% Tween at room temperature. Ileum sections were incubated with anti-WGA-FITC antibody diluted 1:100 with blocking buffer at room temperature for 1 hour, whereas ankle sections were stained with anti-endomucin antibody (Abcam; 1:500), followed by goat anti-rat AF488 (1:1000) under the same condition. Where indicated, ileum and ankle sections were stained with anti-myeloperioxidase (Abcam; 1:200) or anti-IBA1 antibody (Fujifilm; 1:250) followed by anti-rabbit secondary antibodies conjugated to Alexa Fluor 488 and 647 (Invitrogen; 1:1000). Where indicated, spleen sections were stained with anti-IBA1 antibody (Fujifilm; 1:250) and anti-B220 (Biolegend; 1:100) followed by anti-rabbit and anti-rat secondary antibodies conjugated to Alexa Fluor 488 and 647 (Invitrogen; 1:1000).

For immunofluorescence on FFPE mouse ileum and colon sections, heat-induced epitope retrieval was conducted at 110 °C for 15 minutes using citric acid antigen retrieval buffer (1:10; Sigma-Aldrich) in a Decloaking Chamber™ NxGen (Biocare Medical). Then, tissue sections were blocked with 1% BSA in PBS containing 0.2% Triton X-100 and 0.05% Tween at room temperature for 1 hour, followed by anti-ZO-1 (1:200; Cell Signalling) and anti-E-cadherin (1:250; BD Biosciences). Then the sections were stained with goat anti-mouse Alexa Fluor 594 (1:500; Invitrogen) and goat anti-rabbit Alexa Fluor 647 (1:500; Invitrogen) secondary antibodies.

For immunofluorescence on frozen spleen sections, mouse spleens were snap-frozen, embedded in OCT, and stored in -80°C freezer until sectioning. 5 μm spleen sections were obtained using Leica Cryostat CM1950 and fixed with 4% paraformaldehyde. Then sections were blocked at room temperature with 10% goat serum, 1% BSA, 0.2% Triton X-100 and 0.05% Tween diluted in PBS. Anti-F4/80 (1:350; Abcam) or anti-B220 (1:100; Biolegend) primary antibodies were incubated, before goat anti-rat Alexa Fluor 488 (1:500; Invitrogen) secondary antibodies.

All tissue sections were stained with DAPI (1:10000; Sigma-Aldrich) before cover-slipped with Fluoromount with DAPI (Sigma-Aldrich) and left dry for imaging. Fluorescently labelled slides were scanned on the day after mounting with Olympus VS200 Research Slide Scanner with the Excelitas X-Cite XYLIS light source and iDS VS-304M monochrome camera at 20x magnification. Images captured in 16-bit greyscale then false-coloured to the respective fluorochromes. 40x and 60x confocal images were acquired on Olympus FV3000 microscope.

Fluorescence-activated sorting of live myeloid and T cells for adoptive transfer

Myeloid and T cells were sorted from single cell suspensions from spleen and bone marrow samples prepared per above methods. Cells were labelled with LIVE/DEAD™ Aqua (Invitrogen) and CD19-BUV737, CD45.2-BV421, I-A/I-E-BV786, TCRβ-FITC, Ly6G-PerCP-Cy5.5, CD11b-PE, and F4/80-

APC-Cy7 antibodies (Biolegend). Live CD45.2$^+$ TCRb$^-$ CD19$^-$ ClassII$^+$ CD11b$^+$ F4/80$^+$ Ly6G$^-$ macrophages and live CD45.2$^+$ TCRβ$^+$ CD19$^-$ ClassII$^-$ CD11b$^-$ F4/80$^-$ Ly6G$^-$ T cells were sorted using FACS Aria Fusion into foetal bovine serum (FBS). Freshly sorted macrophages are incubated with DiR (Molecular probes) in the dark for 30 min. CD4$^+$ T cells were enriched from spleen and lymph nodes using the CD4$^+$ T cell isolation kit (Miltenyi Biotec) to a purity of 95-97% as determined by flow cytometry. 5x10^5 myeloid cells were injected s.c. to each hock or 5x10^5 CD4 T cells were injected i.v. to SKG mice. Mice were then monitored for clinical scores up and IVIS imaging to 21-35 days post-injection.

Epifluorescence live imaging

Fluorescence imaging (FI) was conducted on an IVIS Spectrum (Revvity, USA) to visualise the location of injected DiR+ macrophages cells at regular time points up to 35 days post-injection, and Luc+ T cells up to 21 days post-curdlan.

To visualise myeloid cells via hock injection, mice were anaesthetised then placed inside the imaging chamber in a prone position with hindjoints stretched and injection sites facing the camera. DiR signals were captured with excitation at 750 nm and emission at 780 nm. At day 7, one animal from each group (SKG mice received myeloid cells from SKG or BALB/c mice, or non-injected) was euthanised, with their spleen, axillary, brachial, mesenteric, lumbar-aortic, inguinal and popliteal lymph nodes, ankle and tail collected and imaged *ex vivo* for DiR+ cells.

To trace i.v.-injected Luc+ T cells, mice were anaesthetised and placed inside the imaging chamber in a supine position. D-luciferin Potassium Salt (Sigma-Aldrich) was administered i.p. 15 min before imaging. The ventral and splenic surface were imaged with IVIS Spectrum (Revvity, USA) up to 21 days post-curdlan.

Brightfield Scanning and Histological Analysis

Clinical features in the mice were monitored weekly and scored by the same observer, who was blinded with regard to treatment, as follows: 0 _ no swelling or redness, 1 _ slight swelling and/or

redness of digits, 2 _ mild swelling and/or redness of footpad, joints or digits, 3 _ severe redness, swelling of the whole paw and multiple digits, and 4_ maximal swelling of paw and joint, ankylosed digits. The scores of the affected joints were summed; the maximum possible score was 16.

Histologic features of the mouse joint were scored on a scale of 1–5, where 1 _ few infiltrating immune cells, 2 _1–2 small patches of inflammation, 3 _ inflammation throughout the ankle joint, 4 _ inflammation in soft tissue/entheses/fasciitis and 5_massive swelling accompanied with inflammation.

The mouse intestine was scored according to three added up criteria: inflammatory infiltrate (ranked 0_absent, 1_scattered, 2_ increased, 3_transmural), granulomata (ranked 0_absent, 1_cell aggregation, 2_ one definite granuloma, 3_multiple granulomata) and villous distortion (ranked 0_absent, 1_height variance of 1/3 of normal, 2_ height variance of 2/3 of normal, 3_ height variance of more than 2/3 of normal). This score was multiplied by the affected cross-sectional area (ranked 0.5_<1%, 1_1-25%, 2_ 26-50%, 3_51-75% and 4_>75%).

Microscopy Image Analysis

OlyVIA and QuPath were used to isolate IHC and fluorescent images. Goblet cells in the ileum were quantified by counting the total number of goblet cells from the crypt–villous junction to the top of 3 villi in 10 different regions of interest (ROI) across the ileum. The average number of positive goblet cells per crypt–villus unit was recorded.

Fluorescent images acquired from the slide scanner and confocal microscopy were visualised and processed using OlyVIA and QuPath.

The ImageJ software was used to quantify bacteria penetration in carnoy-fixed FISH-labelled ileum tissues. At least three areas from each ileum section were selected and exported using QuPath as OME TIFF files before importing into ImageJ. At least ten ROIs outlining the border of three consecutive villi whilst excluding the villi crypts were manually selected. Then, the number of EUB338$^+$ (Cy3 channel) signals within each ROI were counted using the “threshold” function within ImageJ and then normalised to the area of each ROI.

Co-localisation of bacterial DNAs and immune cells were quantified with TissueGnostics's StrataQuest 7.1.138. Fluorescent scanned images were imported into the software. Initially the nuclei were segmented on DAPI channel using Deep Neural Network (DNN) model for nuclear segmentation. Following the nuclear segmentation, a secondary cellular mask was created around nuclei using Cy5 channel to isolate neutrophils (MPO) and macrophages (IBA-1), and colocalization with bacterial DNAs was measured using the presence of Cy3 marker in the proximity of the Cy5 cellular mask. The output dot plots were created within the software and the subsequent cut offs were set using the isotype-matched negative control and internal controls. The results were visually evaluated creating a direct forward and backward connection between the images and the dot plot (red contours for Cy3-Cy5 double positive signal for colocalisation of bacterial DNA with neutrophils or macrophages. The results were exported from the report generator module within the software and used for statistical analysis.

Analysis for Epifluorescence Live Imaging

Fluorescence signals were analysed using IVIS software v4.7.3 (Revvity, USA).

To quantify the epifluorescence of injected myeloid cells, a region of interest (ROI) was drawn over each injection site across all time points for injection of myeloid cells, and the radiant efficiency was calculated. The ratio of radiant efficiency against non-injected joints was calculated, with standard error of the mean compared between groups at days 7, 14, 21, and 28 post-injection. Area under the curve (AUC) was calculated and one-sample t-test of AUC was used to determine statistical significance.

To quantify the bioluminescent of Luc^+ $CD4^+$ T cells, circular ROIs were drawn around the paws and the lymphoid organs to measure signal intensity (by total flux, photons per second) from the organs. Mean ± SEM was compared between 0, 3, 7, 10, 14 and 21 days post-curdlan. Significant differences over time and between curdlan treated mice and naïve mice receiving SKG.luc T cells are determined using two-way ANOVA with Tukey's multiple comparison test.

Supplemental Table 1. Study Resources

REAGENT or RESOURCE	SOURCE	IDENTIFIER
Antibodies		
Brilliant Violet 421™ anti-mouse CD3 (clone 17A2)	Biolegend	Cat# 100228
BUV496 anti-mouse CD4 (clone GK1.5)	BD Biosciences	Cat# 612952
APC anti-mouse CD8a (clone 53-6.7)	Biolegend	Cat# 100712
PE/Cyanine7 anti-mouse/human CD11b (clone M1/70)	Biolegend	Cat# 101216
PE anti-mouse/human CD11b (clone M1/70)	Biolegend	Cat# 101208
Brilliant Violet 605™ anti-mouse/human CD11b (clone M1/70)	Biolegend	Cat# 101257
BUV395 anti-mouse CD11c (clone HL3)	Biolegend	Cat# 564080
Brilliant Violet 605™ anti-mouse CD11c (clone N418)	Biolegend	Cat# 117333
PE/Cyanine7 anti-mouse CD11c Antibody (clone 117318)	Biolegend	Cat# 117318
Brilliant Violet 421™ anti-mouse CD19 (clone 6D5)	Biolegend	Cat# 115549
BUV737 anti-mouse CD19 (clone 1D3)	BD Biosciences	Cat# 612781
BUV395 anti-Mouse CD44 (clone IM7)	BD Biosciences	Cat# 740215
Brilliant Violet 421™ anti-mouse CD45.2 (clone 104)	Biolegend	Cat# 109832
Purified anti-mouse/human CD45R/B220 (clone RA3-6B2)	Biolegend	Cat# 103202
PE/Dazzle™ 594 anti-mouse/human CD45R/B220 (clone RA3-6B2)	Biolegend	Cat# 103258
FITC anti-mouse CD64 (FcγRI) (clone X54-5/7.1)	Biolegend	Cat# 139316
Brilliant Violet 785™ anti-mouse CD86 (clone GL-1)	Biolegend	Cat# 105043
PE/Cyanine7 anti-mouse CD206 (MMR) (clone C068C2)	Biolegend	Cat# 141720
PE anti-mouse CX3CR1 (clone SA011F11)	Biolegend	Cat# 149006
Purified Mouse Anti-E-Cadherin (clone 36/E-cadherin)	BD Biosciences	Cat# 610181
Anti-Endomucin (clone V.7C7.1)	Abcam	Cat# ab106100
Anti-F4/80 antibody (clone CI:A3-1)	Abcam	Cat# ab6640
Alexa Fluor® 647 anti-mouse F4/80 (clone BM8)	Biolegend	Cat# 123122
APC/Cyanine7 anti-mouse F4/80 (clone BM8)	Biolegend	Cat# 123118
FOXP3 monoclonal antibody, Alexa Fluor™ 700 (clone FJK-16s)	Invitrogen	Cat# 56-5773-82
FITC anti-mouse I-A/I-E (clone M5/114.15.2)	Biolegend	Cat# 107606
BV480 anti-mouse I-A/I-E (clone M5/114.15.2)	BD Biosciences	Cat# 566086
Brilliant Violet 785™ anti-mouse I-A/I-E (clone M5/114.15.2)	Biolegend	Cat# 107645
Anti-IBA-1 for Immunocytochemistry	Fujifilm	Cat# 019-19741
Alexa Fluor® 488 anti-mouse IFN-γ (clone XMG1.2)	BD Biosciences	Cat# 557724
PE anti-mouse IL-17A (clone TC11-18H10.1)	Biolegend	Cat# 506904
PerCP/Cyanine5.5 anti-mouse Ly-6G (clone 1A8)	Biolegend	Cat# 127616
APC/Cyanine7 anti-mouse Ly-6C (clone HK1.4)	Biolegend	Cat# 128026
PE-CF594 anti-mouse MerTK (Mer) (clone BB14-16)	BD Biosciences	Cat# 570326
Anti-Myeloperoxidase (clone EPR20257)	Abcam	Cat# ab208670
Brilliant Violet 785™ anti-mouse TCR β chain (clone H57-597)	Biolegend	Cat# 109249
Pacific Blue™ anti-mouse TCR β chain (clone H57-597)	Biolegend	Cat# 109226
FITC anti-mouse TCR β chain (clone H57-597)	Biolegend	Cat# 109206
ZO-1 Rabbit mAb (clone D6L1E)	Cell Signalling	Cat# 13663S
LIVE/DEAD™ Fixable Aqua Dead Cell Stain Kit	Invitrogen	Cat# L34966
BD Horizon™ Fixable Viability Stain 440UV	BD Biosciences	Cat# 566332
Flow-Count Fluorospheres	Beckman Coulter	Cat# 7547053
Lectin from *Triticum vulgaris* (WGA, FITC Conjugate)	Sigma-Aldrich	Cat# L4895
Goat anti-Rabbit IgG (H+L) Highly Cross-Adsorbed Secondary Antibody, Alexa Fluor™ 647	Invitrogen	Cat# A21245
Goat anti-Rat IgG (H+L) Cross-Adsorbed Secondary Antibody, Alexa Fluor™ 488	Invitrogen	Cat# A11006
Bacterial and virus strains		
Lactobacillus murinus	Mark Morrison	ASF361
Parabacteroides goldsteinii	Mark Morrison	ASF519
Altered Schaedler Flora	Mark Morrison	ASF
Chemicals, peptides, and recombinant proteins		
Curdlan	Wako	Cat# 032-09902

Anti-IL23p19 Antibody for Mouse	Eli Lilly	Cat# LSN2479016
Collagenase D	Roche	Cat# 11088866001
DNase 1	Roche	Cat# 11284932001
Hyaluronidase	Sigma	Cat# H3506
Steady-Glo® Luciferase Assay System	Promega	Cat# E2510
Sensifast cDNA synthesis kit	Bioline	Cat# BIO-65054
SensiFAST SYBR Lo-ROX Kit	Bioline	Cat# BIO-94020
Fluoroshield™ with DAPI	Sigma-Aldrich	Cat# F6057
DiOC$_{18}$(7) ('DiR')	Invitrogen	Cat# D1273
Triton™ X-100	Sigma-Aldrich	Cat# X100
Bovine Serum Albumin	Bovogen Biologicals	Cat# BSAS 0.1
Percoll	GE Healthcare	Cat# GEHE17-0891-01
SDS	Sigma-Aldrich	Cat# 74255
Hi-DiTM Formamide	ThermoFisher	Cat# 4311320
Periodic acid	Sigma-Aldrich	Cat# P7875
Schiff's Reagents	Sigma-Aldrich	Cat# 3952016
Alcian Blue 8GX	Sigma-Aldrich	Cat# A3157
Haematoxylin, Gill No.2	Sigma-Aldrich	Cat# GHS216
TRIzolTM Reagent	Invitrogen	Cat# 15596018
Xylene	Point of Care Diagnostics Australia	Cat# XYL10P
100% Ethanol	ChemSupply Australia	Cat# EA043
Ethylenediamine tetra acetic acid disodium salt dehydrate	ChemSupply Australia	Cat# EA023
Sodium Hydroxide Pellet	ChemSupply Australia	Cat# SA178
Paraformaldehyde 4%	ProSciTech	Cat# C005
Formalin solution, neutral buffered, 10%	Sigma-Aldrich	Cat# HT501128
Paraplast for Paraffin	Leica	Cat# 39602012
Steady-Glo	Promega	Cat# E2510
Citrate Buffer 10x Antigen Retriever	Sigma-Aldrich	Cat# C9999
DAPI	Sigma-Aldrich	Cat# D9542
Isoflurane solution	Covetrus	Cat# 029404
D-Luciferin potassium salt	Sigma-Aldrich	Cat# 50227
Resazurin sodium salt	Sigma-Aldrich	Cat# 195481
Vitamin K1	Sigma-Aldrich	Cat# V3501
PBS	Gibco	Cat# 14190250
EDTA	Sigma-Aldrich	Cat# E9884
DL-Dithiothreitol	Sigma-Aldrich	Cat# D0632
Cell Activation Cocktail (without Brefeldin A)	Biolegend	Cat# 423302
eBioscience™ Monensin Solution	ThermoFisher	Cat# 00-4505-51
eBioscience™ Mouse Regulatory T Cell Staining Kit #1	ThermoFisher	Cat# 88-8111-40
Brilliant Stain Buffer	BD Biosciences	Cat# 563794
Experimental models: Cell lines		
Human: Caco-2 cells	ATCC	Cat# CL-188
Experimental models: Organisms/strains		
BALB/c	Purchased from Australia Research Council (SPF), or bred in house (GF)	
SKG	Bred in-house	
BALB/c Luc+	Geoffrey Hill MD	
SKG Luc+	Bred in-house	
Media		
RPMI-1640	ThermoFischer	Cat# 21870-076
BD BACTO™ Brain Heart Infusion (BHI)	BD Biosciences	Cat# 237500
M.R.S. Broth (Dehydrated)	Oxoid	Cat# CM0359

Oligonucleotides		
Target gene	*Forward Primer (5'-3')*	*Reverse Primer (5'-3')*
Mouse-*Il23a*	AGCGGGACATATGAATCTACTAAGAGA	GTCCTAGTAGGGAGGTGTGAAGTTG
Mouse-*Il6*	GAGGATACCACTCCCAACAGACC	AAGTGCATCATCGTTGTTCATACA
Mouse-*Tnf*	CATCTTCTCAAAATTCGAGTGACAA	TGGGAGTAGACAAGGTACAACCC
Mouse-*Hspa5*	TGCTGCTAGGCCTGCTCCGA	CGACCACCGTGCCCACATCC
Mouse-*Xbp1*	GAGTCCGCAGCAGGTGC	CAAAAGGATATCAGACTCAGAATCTGAA
Mouse- *Cxcl2*	ACCACCAGGCTACAGGGGCT	GGTCCTGGGGGCGTCACACT
Mouse-*Reg3g*	ACTCCCTGAAGAATATACCCTCC	CGCTATTGAGCACAGATACGAG
Mouse-*Reg3b*	GGCCATATCTGCATCATACCAG	ATGCTTCCCCGTATAACCATCA
Mouse-*Il18*	ACGTGTTCCAGGACACAACA	ACAAACCCTCCCCACCTAAC
Mouse-*Il10*	GGTTGCCAAGCCTTATCGGA	ACCTGCTCCACTGCCTTGCT
Mouse-*Il1b*	CAACCAACAAGTGATATTCTCCATG	GATCCACACTCTCCAGCTGCA
Mouse-*Cldn4*	GGCGTCTATGGGACTACAGG	GAGCGCACAACTCAGGATG
Mouse-*Cldn8*	GTGGATGTGGCCCTAAAGC	CGCTGTGGTCCAGCCTAT
Mouse-*Cldn1* [28]	TCCTTGCTGAATCTGAACA	AGCCATCCACATCTTCTG
Mouse-*Cldn2* [28]	TATGTTGGTGCCAGCATTGT	TCATGCCCACCACAGAGATA
Mouse-*Cldn15* [28]	GCTTCTTCATGTCAGCCCTG	TTCTTGGAGAGATCCATGTTGC
Mouse-*Ocln* [28]	CCTCCAATGGCAAAGTGAAT	CTCCCCACCTGTCGTGTAGT
Mouse-*Tjp1* [28]	CCACCTCTGTCCAGCTCTTC	CACCGGAGTGATGGTTTTCT
Mouse-*Hp*	CTGTCGCTGAGTATGGTGTG	ATGTGCAGCCTTCTAGTGAG
Mouse-*Muc2*	CCATTGAGTTTGGGAACATGC	TTCGGCTCGGTGTTCAGAG
ASF361	GCAATGATGCGTAGCCGAAC	GCACTTTCTTCTCTAACAACAGGG
ASF519	CACAGTAAGCGGCACAGCG	CCGCTCACACGGTAGCTG
ASF502	CGGTACCGCATGGTACAGAGG	CAATGCAATTCCGGGGTTGG
ASF457	CCGAAAGGTGAGCTAATGCCGG	GGGACGCGAGTCCATCTTTC
ASF492	CTGCGGAATTCCTTCGGGG	CCCATACCACCGGAGTTTTC

Bacterial DNA Probes	
EUB338	/5Cy3/GCT GCC TCC CGT AGG AGT
Non-Sense EUB338	/56-FAM/ACT CCT ACG GGA GGC AGC

Software and algorithms		
GraphPad Prism 9	GraphPad	graphpad.com
FlowJo v9	BD	flowjo.com
OlyVIA	Olympus	OlyVIA 3.1
QuPath	QuPath	0.4.1
StrataQuest	TissueGnostics	7.1.138
ImageJ	NIH	1.54h
IVIS Software	PerkinElmer	v4.7.3
Adobe Illustrator CS6	Adobe Inc.	N/A

Other		
BD LSRFortessa™ Cell Analyser	BD	LSR2
Olympus VS120 Brightfield Slide Scanner	Olympus	VS100
Olympus VS200 Fluorescent Slide Scanner	Olympus	VS200
Olympus FV3000 Laser Scanning Confocal Microscope	Olympus	FV3000
Decloaking Chamber™ NxGen	Biocare Medical	DC 2012
Hand homogeniser IKA	IKA	3240000
Precellys 24 Touch Homogeniser	Bertin Technology	P002391-P24T0
Leica Cryostat CM1950	Leica	CM1950
Tissue-Tek Prisma® Plus Automated Slide Stainer	Sakura	6170
Tissue-Tek VIP®6 AI Tissue Processor	Sakura	6040
Stinger anaesthesia machine	Darvallvet	2848
IVIS Spectrum CT system	PerkinElmer	-
Graphical Abstract	Created with BioRender.com	

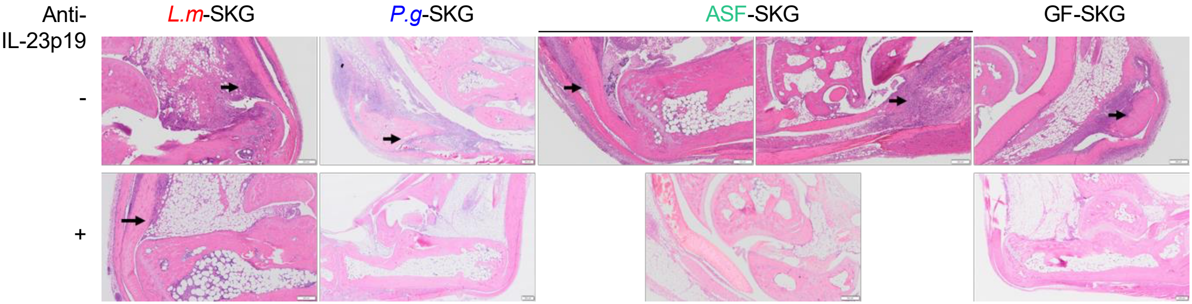
Anti-
IL-23p19
L.m-SKG
P.g-SKG
ASF-SKG
GF-SKG
-
+

Supplemental Figure 1. Mono-association with *P. goldsteinii* and *L. murinus* is sufficient for curdlan-induced ileitis and arthritis, related to Figures 1 and 2.

GF-SKG mice were mono-colonised with *L. murinus*, *P. goldsteinii* or ASF 4 weeks prior to curdlan i.p. at day 0, with n=4-9 across 3 independent experiments. Some mice were treated with anti-IL-23p19 at day -1. Mice were monitored for 5 weeks.
Magnification of the H&E analysis for the rear ankle as shown in **Figure 1B**. Arrows point to areas of immune infiltration. Scale bar is 200 μm.

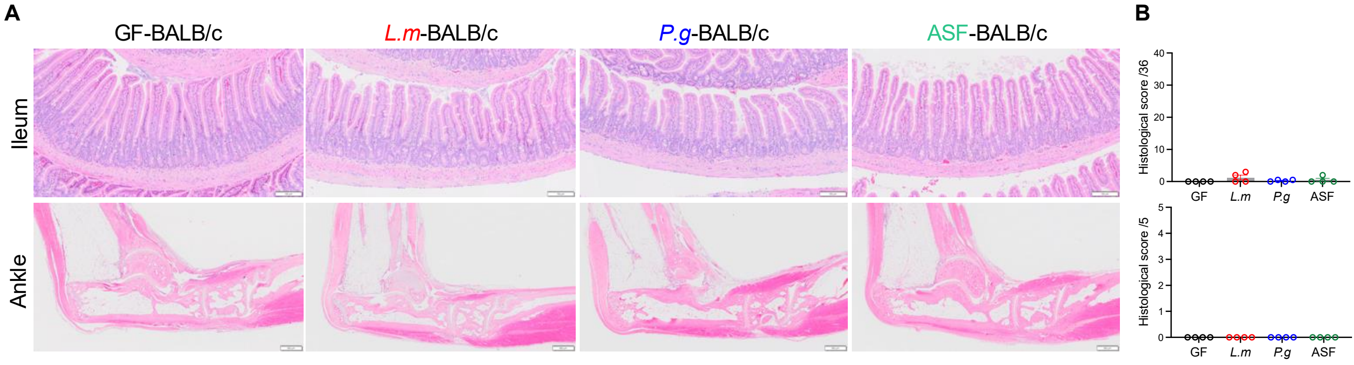
A
GF-BALB/c
L.m-BALB/c
P.g-BALB/c
ASF-BALB/c
Ileum
Ankle
B
Histological score /36
Histological score /5
GF
L.m
P.g
ASF

Supplemental Figure 2. Monocolonised BALB/c mice are resistant to gut and joint disease post-curdlan, related to Figures 1 and 2**.**

GF-BALB/c mice were orally mono-colonised with *L.m*, *P.g* or ASF 4 weeks prior to curdlan i.p. at day 0, n=4 in 1 experiment, per experimental design shown in **Figure 2A**. Mice were monitored for 5 weeks.
(A) Representative H&E staining of ileum with scale bar at 100 μm (**top**) and rear ankle with scale bar at 500 μm (**bottom**).
(B) Cumulative histological score at 5 weeks post curdlan of ileitis, and peripheral arthritis.
Data show mean +/- SEM.

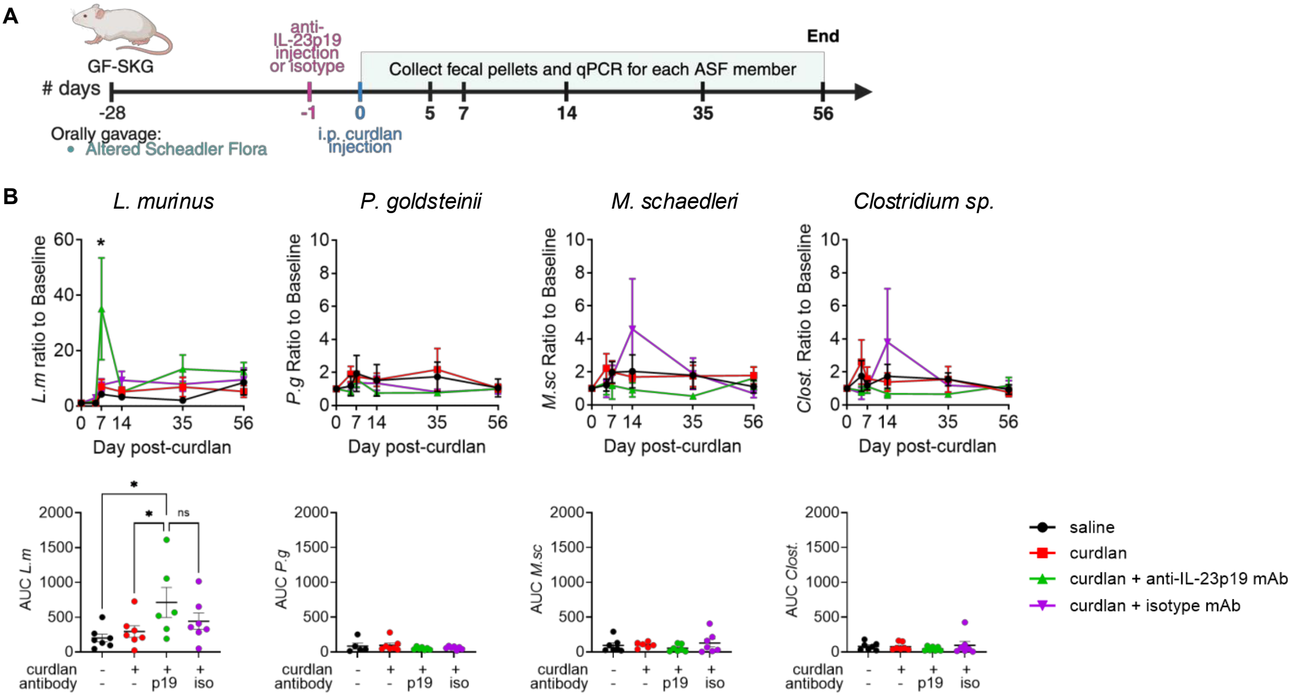
A
GF-SKG
anti-IL-23p19 injection or isotype
End
Collect fecal pellets and qPCR for each ASF member
days
-28
-1
0
5
7
14
35
56
Orally gavage:
Altered Scheadler Flora
i.p. curdlan injection
B
L. murinus
P. goldsteinii
M. schaedleri
Clostridium sp.
L.m ratio to Baseline
P.g Ratio to Baseline
M.sc Ratio to Baseline
Clost. Ratio to Baseline
Day post-curdlan
AUC L.m
AUC P.g
AUC M.sc
AUC Clost.
curdlan
antibody
p19
iso
ns
saline
curdlan
curdlan + anti-IL-23p19 mAb
curdlan + isotype mAb

Supplemental Figure 3. *L. murinus* fecal load in vivo is IL-23 dependent.

GF-SKG mice were mono-colonised with the ASF mini-consortium 4 weeks prior to curdlan i.p. at day 0. At day -1, groups of six ASF-SKG mice were treated with anti-IL-23p19, control isotype, curdlan only or saline.
(Top) Data show analysis by qPCR of each of the 4 bacteria species in ileum of ASF-SKG mice at day 0, 5, 7, 14, 35 and 56 post-curdlan.
(Bottom) Corresponding AUC to the data in the top row.
Statistical analysis: Two-way ANOVA with *p<0.5, **p<0.01, ***p<0.001, ****p<0.0001.

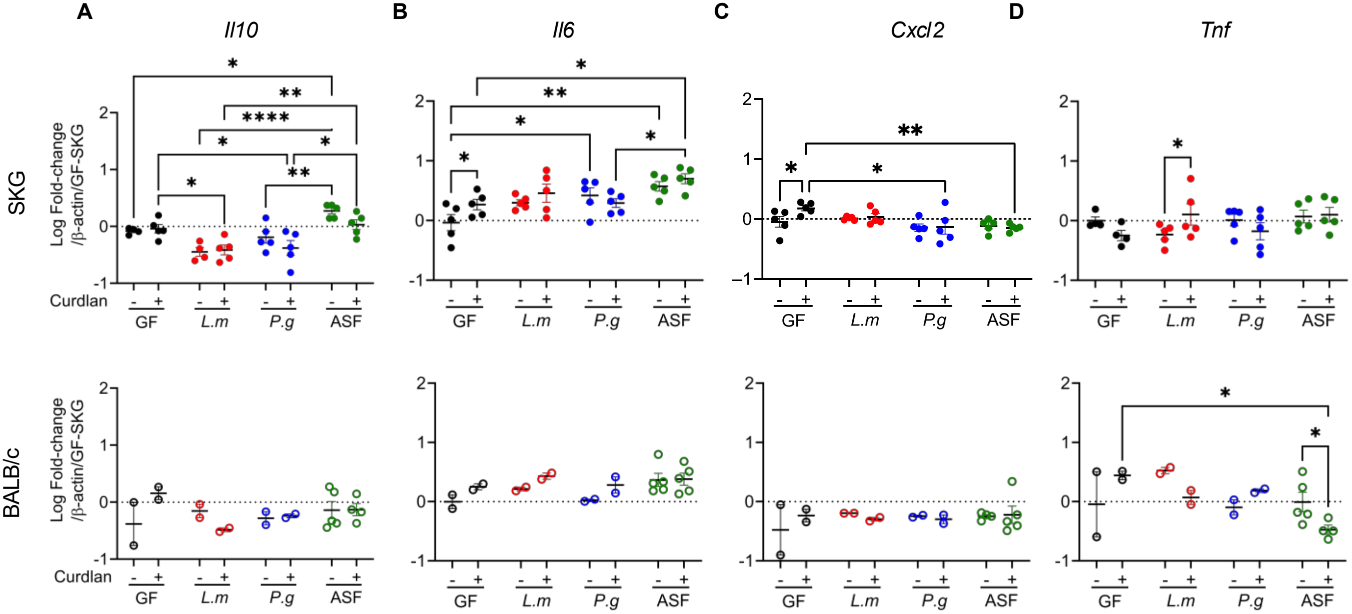

A
Il10
B
Il6
C
Cxcl2
D
Tnf
SKG
BALB/c
Log Fold-change /β-actin/GF-SKG
Curdlan
- +
GF
L.m
P.g
ASF

Supplemental Figure 4. BALB/c mice are resistant to curdlan-induced ileal ER stress, related to Figure 3**.**

GF-SKG and BALB/c mice were mono-colonised with *L. murinus*, *P. goldsteinii* or ASF 4 weeks prior to curdlan i.p. at day 0. Some SKG mice were treated with anti-IL-23p19 at day -1, n=2-5 in across 2 independent experiments per experimental design shown in **Figure 2A**.
Data show log value of the fold increase against naïve GF-SKG for the expression of **(A)** *Il-10*, **(B)** *Il-6,* **(C)** *Cxcl2* and **(D)** *Tnf* in SKG **(top)** and BALB/c **(bottom)**.
Data show mean +/- SEM.
Statistical analysis: Two-way ANOVA with *p<0.5, **p<0.01, ***p<0.001, ****p<0.0001.

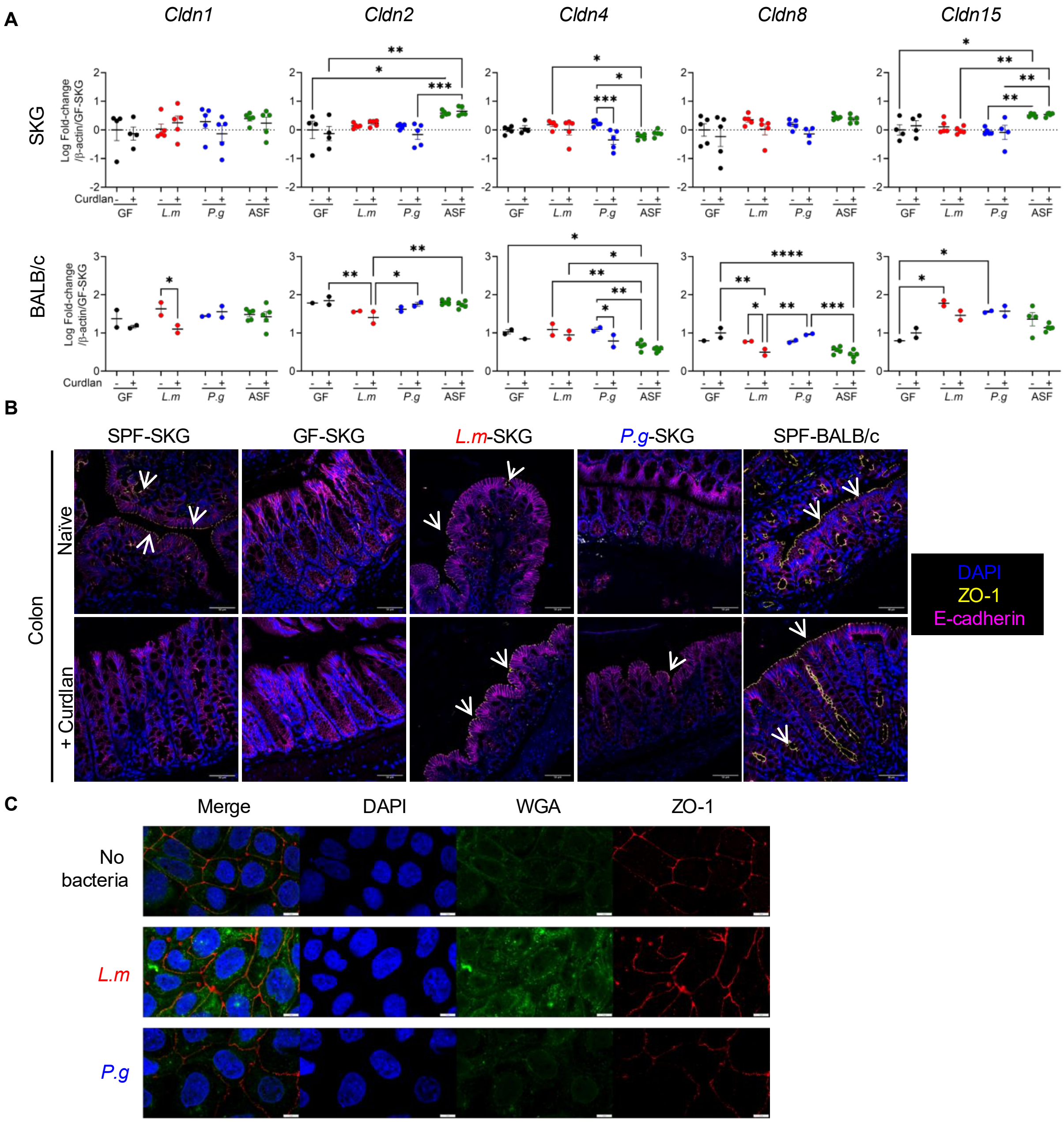
A
Cldn1
Cldn2
Cldn4
Cldn8
Cldn15
SKG
BALB/c
Log Fold-change /β-actin/GF-SKG
Curdlan
GF
L.m
P.g
ASF
B
SPF-SKG
GF-SKG
L.m-SKG
P.g-SKG
SPF-BALB/c
Colon
Naïve
+ Curdlan
DAPI
ZO-1
E-cadherin
C
Merge
DAPI
WGA
ZO-1
No bacteria
L.m
P.g

Supplemental Figure 5. Comparing colonic gut fitness between BALB/c and SKG mice, related to Figures 4 and 5.

GF-BALB/c and GF-SKG mice were monocolonised with *L.m*, *P.g* or ASF at 4 weeks prior to curdlan i.p. at day 0 with n=2-4 across 2 experiments. Mice were sacrificed at day 7 post-curdlan per experimental design shown in **Figure 2A**.

(A) Ileum tissues were assessed for gene expression of the claudin family by qPCR. Data show mean +/- SEM with each data point representing one individual mouse.

(B) Representative confocal images of colonic biopsy stained fluorescently with E-cadherin (magenta), ZO-1 (yellow) and DAPI (blue). Arrows point to ZO-1+ at the apical surface of colonic epithelium. SPF-SKG and BALB/c are used as controls. Scale bars are 50 μm.

(C) Colonic Caco-2 monolayer co-cultured *in vitro* with *L.m* or *P.g* for 4 hrs, stained with WGA-FITC (green), ZO-1 (red) and DAPI (blue) and imaged by confocal microscopy. Scale bars are 10 μm.

Statistical analysis: Two-way ANOVA with **p*<0.5, ***p*<0.01, ****p*<0.001, *****p*<0.0001.

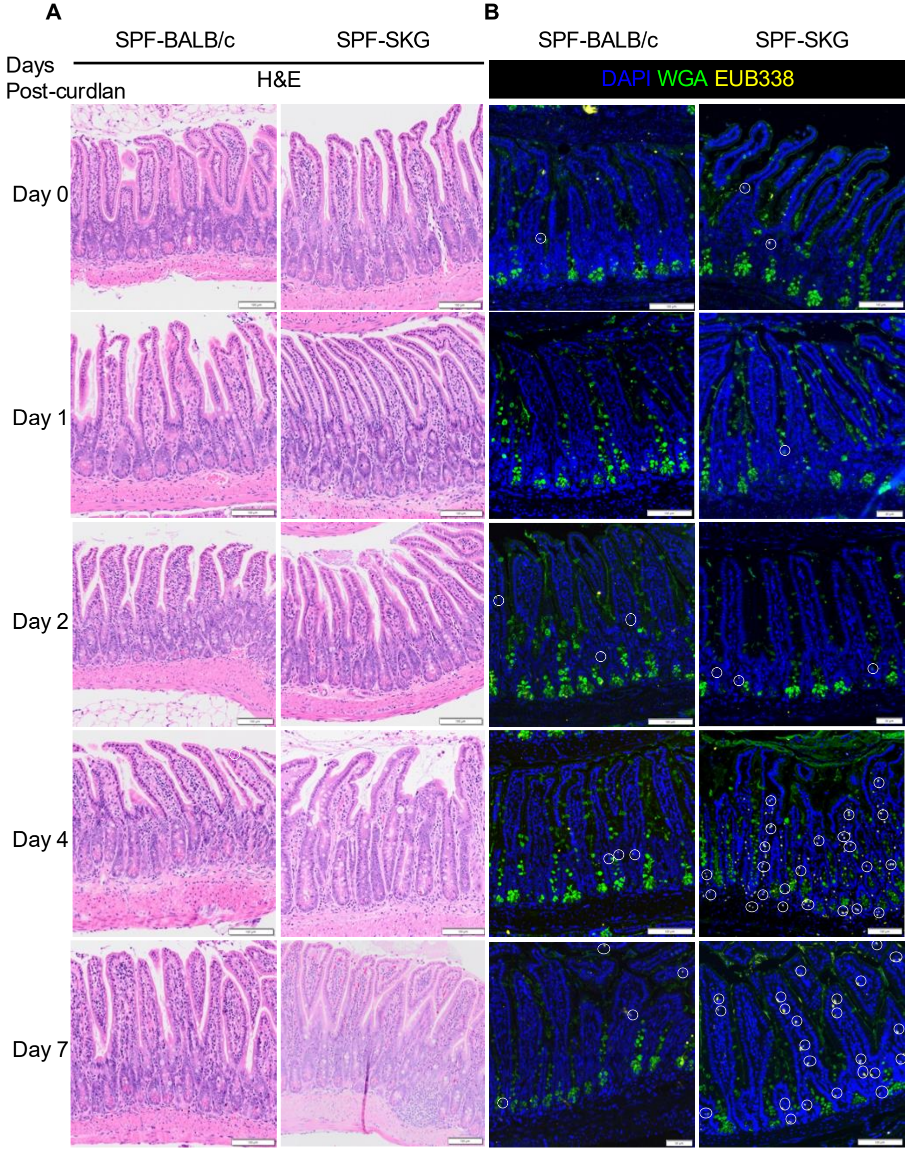

A
B
SPF-BALB/c
SPF-SKG
SPF-BALB/c
SPF-SKG
Days
Post-curdlan
H&E
DAPI WGA EUB338
Day 0
Day 1
Day 2
Day 4
Day 7

Supplemental Figure 6. Gut bacteria invade the gut villi of SPF-SKG and BALB/c mice from day 4 post-curdlan.

SPF-SKG and SPF-BALB/c mice were treated with curdlan i.p. at day 0 and terminal ileum was collected at day 0, 1, 2, 4 and 7 post-curdlan, n=2 per group across 2 independent staining experiments. Representative **(A)** H&E staining and **(B)** immunofluorescence staining DAPI (blue), EUB338 (yellow), WGA-FITC (green). White circles show bacteria. Scale bars are 50 or 100 μm.

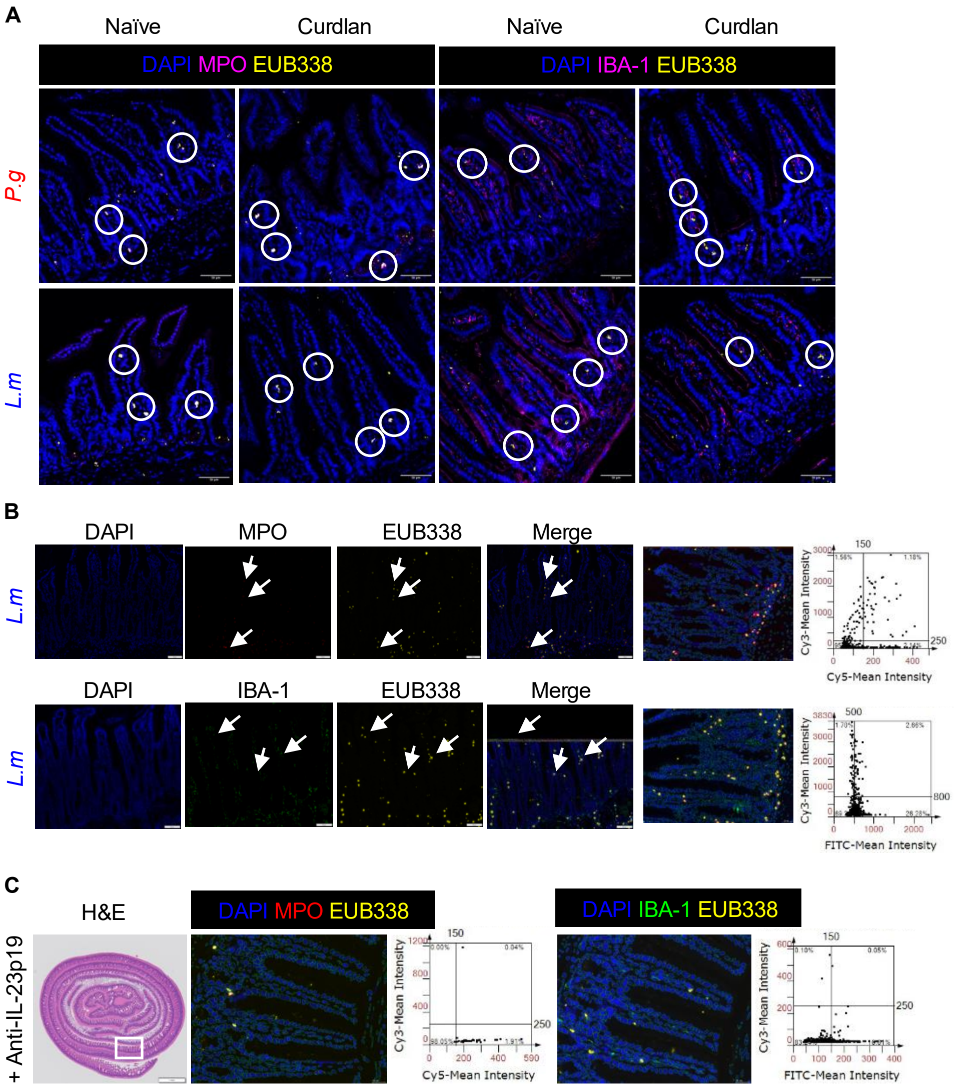
A
Naïve
Curdlan
Naïve
Curdlan
DAPI MPO EUB338
DAPI IBA-1 EUB338
P.g
L.m
B
DAPI
MPO
EUB338
Merge
L.m
Cy3-Mean Intensity
Cy5-Mean Intensity
150
250
DAPI
IBA-1
EUB338
Merge
L.m
FITC-Mean Intensity
500
800
C
H&E
DAPI MPO EUB338
DAPI IBA-1 EUB338
+ Anti-IL-23p19
Cy3-Mean Intensity
Cy5-Mean Intensity
150
250
FITC-Mean Intensity

Supplemental Figure 7. Bacterial DNA associates with neutrophils and macrophages in intestinal villi in a curdlan- and IL-23-dependent manner, related to Figures 6 and 7.

GF-SKG and GF-BALB/c mice remained GF or were monocolonised with *P.g* or *L.m* 4 weeks prior to curdlan i.p. at day 0. Some mice were treated with anti-IL-23p19 at day -1. At 1 and 5 weeks post-curdlan, ileum tissues were assessed for bacterial translocation from lumen to villi by imaging using FISH with EUB338 probe and co-staining with anti-MPO or anti-IBA-1 antibodies, per experimental design shown in **Figure 4A**.

(A) Representative confocal images of *P.g*-SKG and *L.m*-SKG at 1-week post-curdlan, with DAPI (blue), EUB338 (yellow), MPO or IBA-1 (red).

(B) Representative fluorescently labelled confocal images of *L.m*-SKG at 5-weeks post-curdlan, with DAPI (blue), EUB338 (yellow), MPO or IBA-1 (red). Green contours for boundaries of individual cells and its quantifications are shown on the right.

(C) Representative H&E and fluorescently labelled images from anti-IL-23p19-treated *L.m*-SKG, with green contours indicating cell boundaries, DAPI (blue), EUB338 (yellow), MPO (red) or IBA-1 (green).

Scale bars are 50 or 100 μm.

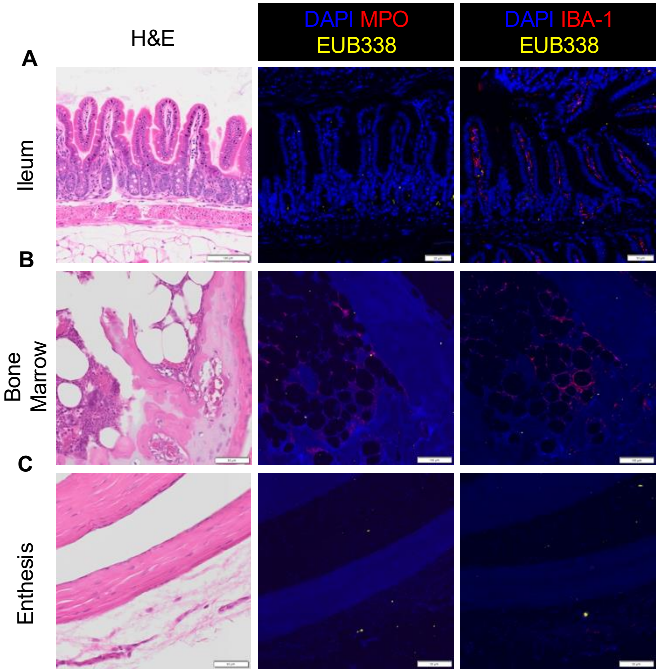
H&E
DAPI MPO
EUB338
DAPI IBA-1
EUB338
A
Ileum
B
Bone
Marrow
C
Enthesis

Supplemental Figure 8. No EUB338+ signals are found in the gut and joint of GF-SKG mice, related to Figure 7.

GF-SKG mice were injected with curdlan injection i.p. at day 0. Ileum and ankle were assessed for bacterial translocation using FISH with EUB338 and anti-MPO or anti-IBA-1 antibodies at 1-week post-curdlan.

Representative H&E, confocal images for **(A)** ileum, **(B)** bone marrow and **(C)** enthesis with DAPI (blue), EUB338 (yellow), and MPO/IBA-1 (magenta), with n=2 per group across 2 independent staining experiments. Scale bars are 50 μm.

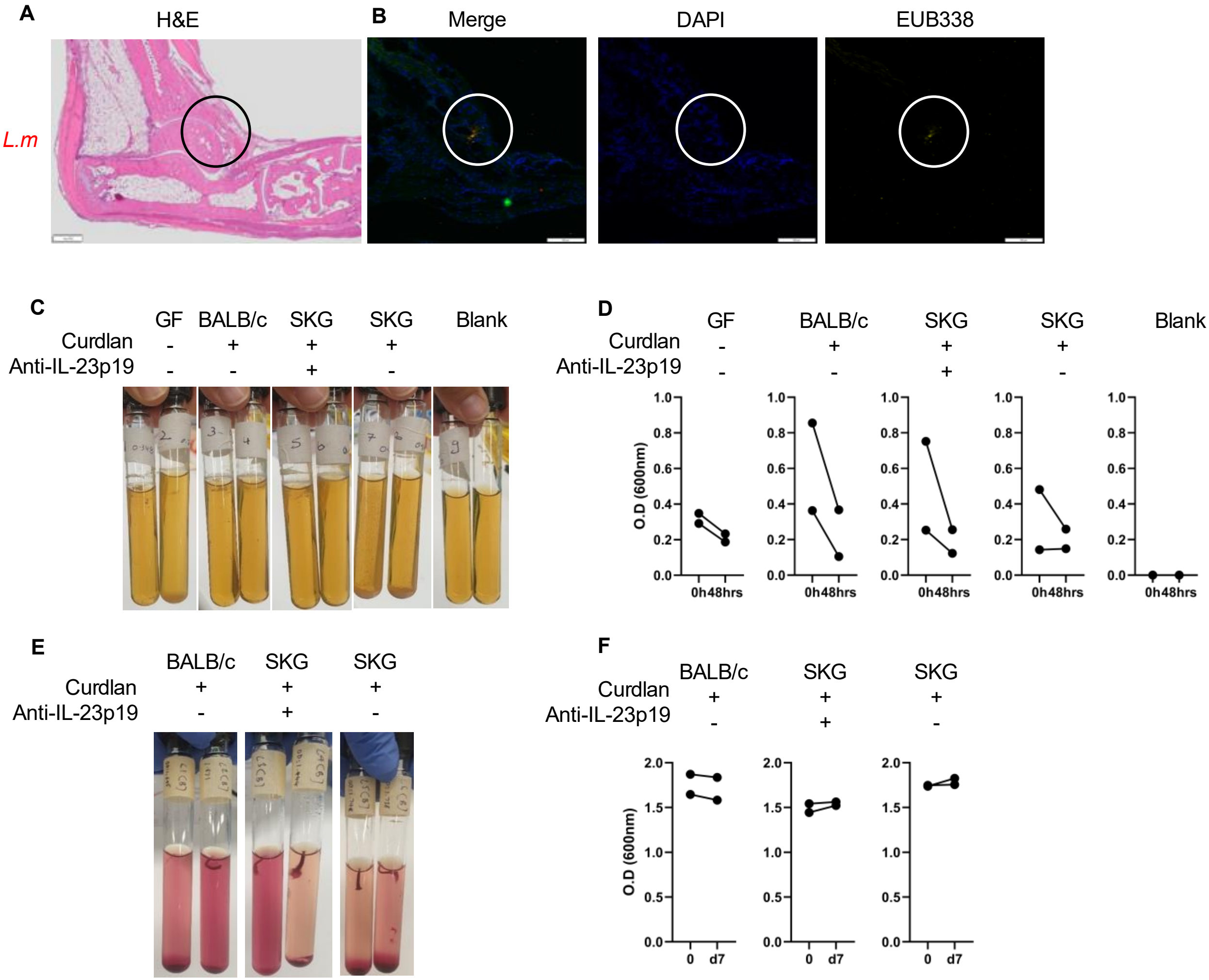
A
H&E
L.m
B
Merge
DAPI
EUB338
C
GF
BALB/c
SKG
SKG
Blank
Curdlan
Anti-IL-23p19
D
O.D (600nm)
0h48hrs
E
F
0
d7

Supplemental Figure 9. Bacterial DNAs from ankle or peripheral blood are not culturable.

GF-SKG and BALB/c mice were mono-colonised with *L.m* 4 weeks prior to curdlan i.p. at day 0. Some SKG mice were treated with anti-IL-23p19 or isotype control at day -1. Ankle and peripheral blood were harvested aseptically and proceeded for bacterial culture in MRS medium as the blank, with n=2 per group in 1 experiment.
Representative H&E with a 500 µm scale bar **(A)** and confocal images with a 100 µm scale bar for **(B)** *L.m*-SKG rear ankle with DAPI (blue), EUB338 (yellow) and endomucin (green).
(C) Crushed ankle culture and **(D)** absorbance O.D. 600nm at 48 hours
(E) Whole blood culture and **(F)** absorbance for O.D. 600nm at 1 week.

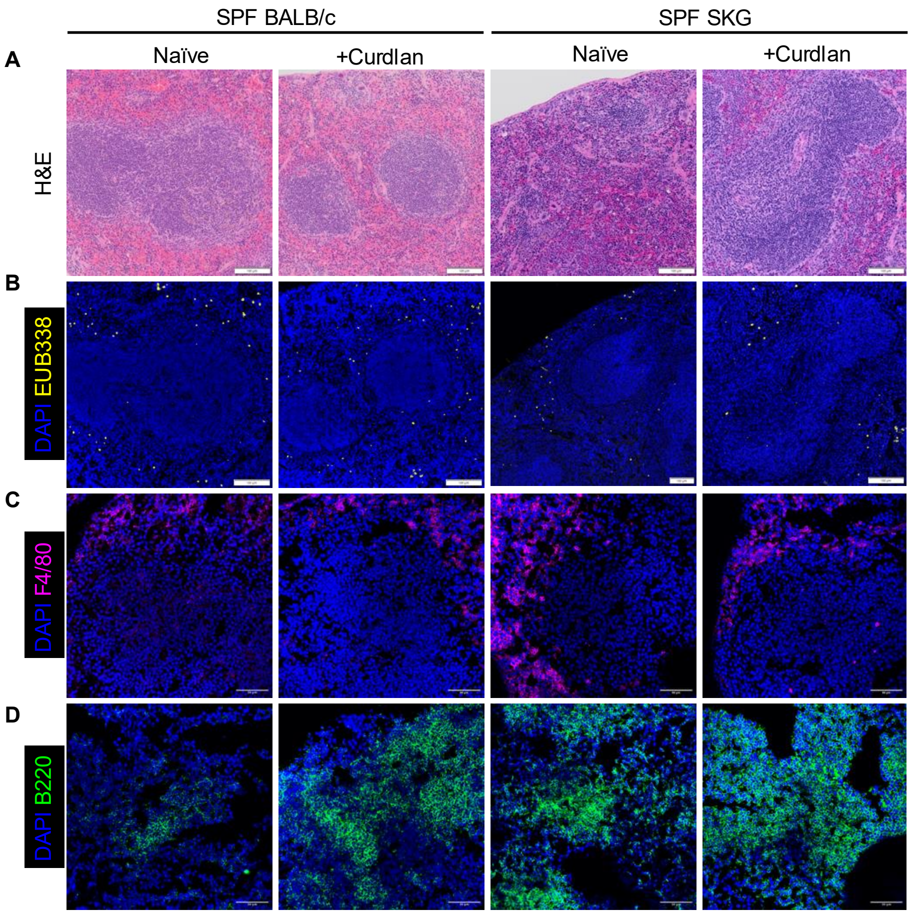
SPF BALB/c
SPF SKG
Naïve
+Curdlan
Naïve
+Curdlan
A
H&E
B
DAPI EUB338
C
DAPI F4/80
D
DAPI B220

Supplemental Figure 10. EUB338$^+$ myeloid cells are present in the splenic red pulp, related to Figure 11**.**

SPF-SKG and SPF-BALB/c mice were injected with curdlan i.p. at day 0 and their spleens were collected at day 7, per experimental design shown in **Figure 7A**. Naïve-SKG or BALB/c mice were used as controls.
Representative **(A)** H&E and confocal imaging of **(B)** EUB338 staining (yellow) with FISH in FFPE spleen sections. Scale bars are 100 µm.
Confocal imaging of **(C)** F4/80 (magenta) and **(D)** B220 (green) immunofluorescent staining of frozen spleen sections showing macrophages outside the B-cell rich white pulp follicles.
Scale bars are 50 µm.

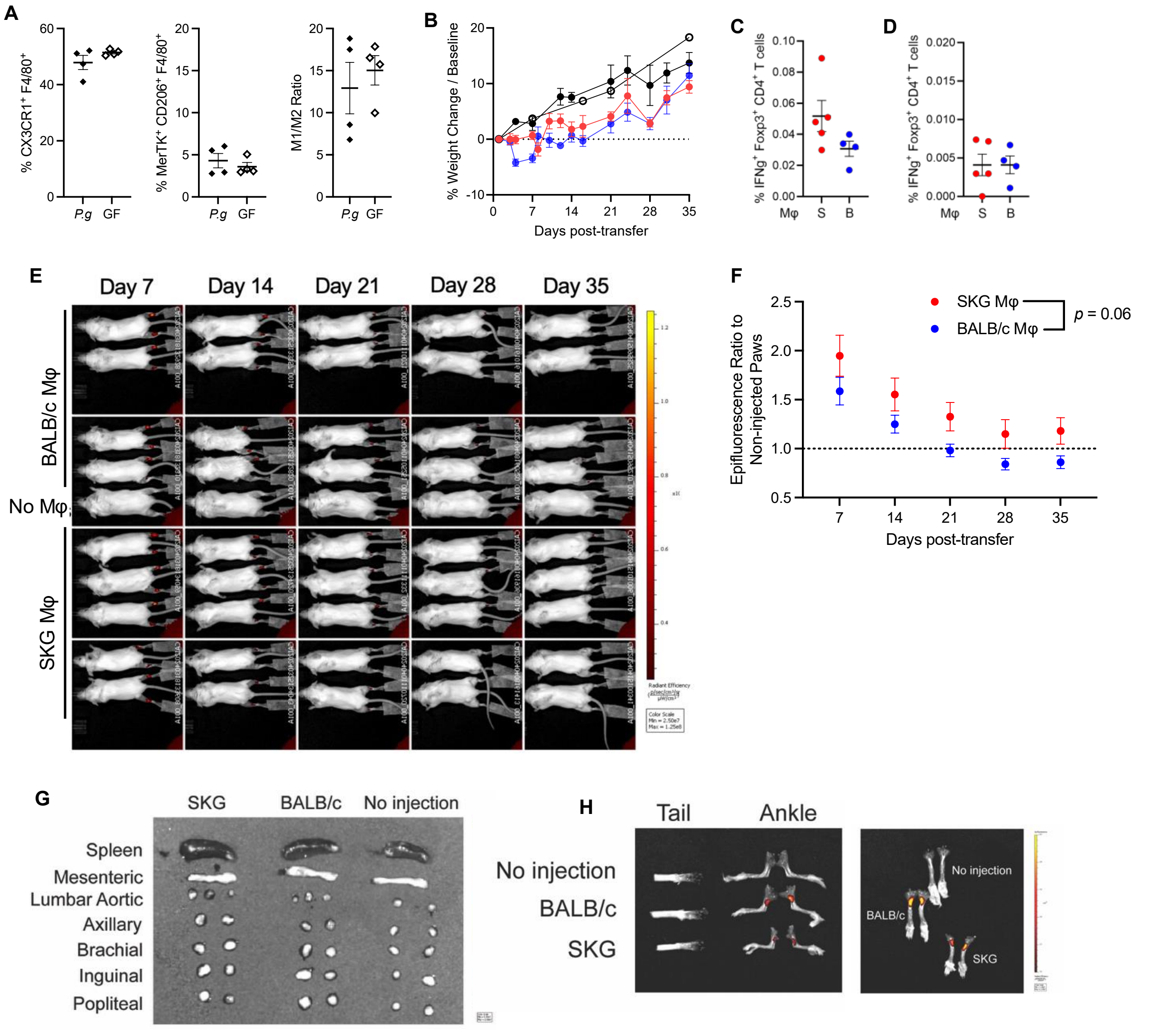
A
% CX3CR1+ F4/80+
P.g
GF
% MerTK+ CD206+ F4/80+
M1/M2 Ratio
B
% Weight Change / Baseline
Days post-transfer
C
% IFNg+ Foxp3+ CD4+ T cells
Mφ
S
B
D
E
Day 7
Day 14
Day 21
Day 28
Day 35
BALB/c Mφ
No Mφ
SKG Mφ
Radiant Efficiency
F
SKG Mφ
BALB/c Mφ
p = 0.06
Epifluorescence Ratio to Non-injected Paws
Days post-transfer
G
SKG
BALB/c
No injection
Spleen
Mesenteric
Lumbar Aortic
Axillary
Brachial
Inguinal
Popliteal
H
Tail
Ankle
No injection
BALB/c
SKG

Supplemental Figure 11. Hock-injected arthritogenic myeloid cells induce local inflammation in naïve SPF-SKG recipients, related to Figures 11 and 12**.**

(A) M1-like, M2-like and M1/M2 ratio from 1-week curdlan-treated *P.g*-SKG compared to GF-SKG as down in **Figure 7H**.

(B-F) $CD45.2^{+}$ $TCR\beta^{-}$ $CD19^{-}$ $CD11b^{+}$ $Ly6G^{-}$ myeloid cells were sorted from the spleen and BM of SPF-SKG, SPF-BALB/c or GF SKG and adoptively transferred to naïve SPF-SKG mice at day 0 (s.c. hock) per experimental design shown in **Figure 7I**, with n=4-5 across 2 independent experiments. SPF-SKG and SPF-BALB/c macrophages were labelled with DiR and traced with IVIS imaging system at indicated timepoints.

(B) Weight changes. Data show mean +/- SEM for the indicated groups.

(C-D) At 35 days post-injection, IFNγ-producing regulatory T cells from the pLN **(C)** spleen **(D)** were analysed by FACS. Data show mean +/- SEM with each data point representing one individual mouse.

(E) Longitudinal tracking of DiR^{+} injected cells with **(F)** quantification of epifluorescence with two ROIs in each mouse for n=4-5 in each group. Data show mean +/- SEM for each group at indicated timepoints.

At day 7 post-transfer, lymphoreticular organs **(G)**, rear ankle and tail **(H)** were collected from 1 mouse of each group to look for DiR luminescent signals.

Statistical analysis: Mann-Whitney test **(B-D, F)**; all analyses were non-significant.
